# Naphthoquinone-Quinolone Hybrids with Antitumor Effects on Breast Cancer Cell Lines—From the Synthesis to 3D-Cell Culture Effects

**DOI:** 10.3390/ijms25126490

**Published:** 2024-06-12

**Authors:** Vanessa da Gama Oliveira, Marcelly Muxfeldt, Mariana Muniz da Paz, Mayra Silva Coutinho, Raissa Eduardo dos Santos, Giulia Diniz da Silva Ferretti, Danielly C. Ferraz da Costa, Pedro Fonseca Regufe, Ivson Lelis Gama, Fernanda da Costa Santos Boechat, Emersom Silva Lima, Vitor Francisco Ferreira, Marcela Cristina de Moraes, Maria Cecília Bastos Vieira de Souza, Pedro Netto Batalha, Luciana Pereira Rangel

**Affiliations:** 1 Instituto Nacional de Infectologia, Fundação Oswaldo Cruz, Rio de Janeiro 21040-900, RJ, Brazil; vanessagama@id.uff.br; 2 Instituto de Química, Universidade Federal Fluminense, Niteroi 24020-141, RJ, Brazil; mayrasc@id.uff.br (M.S.C.); pedroregufe@gmail.com (P.F.R.); ivsonlelis@yahoo.com.br (I.L.G.); fernandacostasantos@id.uff.br (F.d.C.S.B.); mcmoraes@id.uff.br (M.C.d.M.); mceciliabvs@gmail.com (M.C.B.V.d.S.); 3 Faculdade de Farmácia, Universidade Federal do Rio de Janeiro, Rio de Janeiro 21941-902, RJ, Brazil; muxfeldtm@gmail.com (M.M.); mariana.mmp@outlook.com (M.M.d.P.); raissaeduardo1@gmail.com (R.E.d.S.); 4 Faculdade de Ciências Farmacêuticas, Universidade Federal do Amazonas, Manaus 69067-005, AM, Brazil; eslima@ufam.edu.br; 5 Instituto de Bioquimica Médica Leopoldo de Meis, Universidade Federal do Rio de Janeiro, Rio de Janeiro 21941-902, RJ, Brazil; giuliadiniz@hotmail.com; 6 Instituto de Nutrição, Universidade do Estado do Rio de Janeiro, Rio de Janeiro 20550-013, RJ, Brazil; daniellyferraz@ymail.com; 7 Faculdade da Amazônia Legal, Colider 78500-000, MT, Brazil; 8 Faculdade de Farmácia, Universidade Federal Fluminense, Niteroi 24020-141, RJ, Brazil; vitorferreira@id.uff.br

**Keywords:** 4-quinolone, 1,4-naphthoquinone, anticancer, breast cancer

## Abstract

Breast cancer stands as one of the foremost cause of cancer-related deaths globally, characterized by its varied molecular subtypes. Each subtype requires a distinct therapeutic strategy. Although advancements in treatment have enhanced patient outcomes, significant hurdles remain, including treatment toxicity and restricted effectiveness. Here, we explore the anticancer potential of novel 1,4-naphthoquinone/4-quinolone hybrids on breast cancer cell lines. The synthesized compounds demonstrated selective cytotoxicity against Luminal and triple-negative breast cancer (TNBC) cells, which represent the two main molecular types of breast cancer that depend most on cytotoxic chemotherapy, with potency comparable to doxorubicin, a standard chemotherapeutic widely used in breast cancer treatment. Notably, these derivatives exhibited superior selectivity indices (SI) when compared to doxorubicin, indicating lower toxicity towards non-tumor MCF10A cells. Compounds 11a and 11b displayed an improvement in IC_50_ values when compared to their precursor, 1,4-naphthoquinone, for both MCF-7 and MDA-MB-231 and a comparable value to doxorubicin for MCF-7 cells. Also, their SI values were superior to those seen for the two reference compounds for both cell lines tested. Mechanistic studies revealed the ability of the compounds to induce apoptosis and inhibit clonogenic potential. Additionally, the irreversibility of their effects on cell viability underscores their promising therapeutic utility. In 3D-cell culture models, the compounds induced morphological changes indicative of reduced viability, supporting their efficacy in a more physiologically relevant model of study. The pharmacokinetics of the synthesized compounds were predicted using the SwissADME webserver, indicating that these compounds exhibit favorable drug-likeness properties and potential as antitumor agents. Overall, our findings underscore the promise of these hybrid compounds as potential candidates for breast cancer chemotherapy, emphasizing their selectivity and efficacy.

## 1. Introduction

According to the Global Cancer Observatory (GLOBOCAN 2022), in 2022, breast cancer was ranked as the second most prevalent cancer in the world [1]. Breast cancer is diagnosed using a molecular classification that includes four major subtypes based on histopathological findings: Luminal A, Luminal B, HER2 overexpression, and Basal-like or triple-negative breast cancer (TNBC), determined by the expression of progesterone, estrogen, HER2 receptors, and Ki67 levels [2]. This classification guides treatment choices, which typically involve a combination of procedures, such as surgical removal, radiation therapy, and chemotherapy, including adjuvant/neoadjuvant chemotherapy and targeted therapy with antibodies, depending on the specific case.

Systemic therapies are critical for controlling micrometastases and preventing their spread throughout the body. Treatment approaches vary based on the type of tumor cells present. Classic cytotoxic chemotherapy agents include doxorubicin (adriamycin), cyclophosphamide, taxol, 5-fluorouracil (5-FU), and others. For hormone-responsive cells, adjuvant therapy is employed to target hormone production using aromatase inhibitors such as anastrozole or to block estrogen receptors with drugs such as tamoxifen. HER2+ tumors are treated with targeted therapy such as trastuzumab (herceptin). TNBC has the worst prognosis, with higher recurrence rates and limited treatment options. Also, toxic and side effects are common, such as cardiotoxicity associated with doxorubicin [3]. For this reason, the search for new systemic chemotherapy is still ongoing, with a focus on drugs with lower toxicity to non-tumor cells. Our aim in this work was to describe and compare the effects of novel potential lead compounds for chemotherapy, hybrids of 4-quinolone and 1,4-naphthoquinone, on cancer cell lines that represent two of the most common cancer types (Luminal and TNBC) that benefit from systemic cytotoxic chemotherapy, with an emphasis on higher selectivity for tumor cells over non-tumor cells.

**Figure 1:**
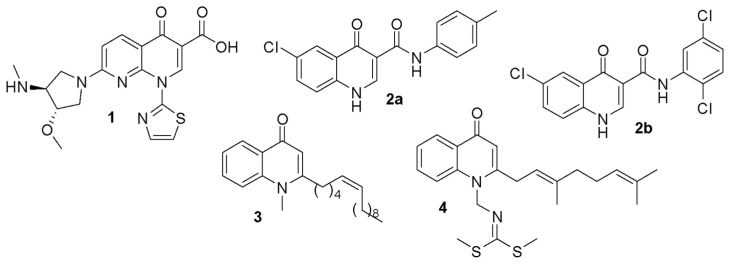
Examples of 4-quinolone derivatives and analogs (**1**–**4**) with anticancer activity.

4-quinolones are a class of heterocyclic compounds widely known as antibacterial agents due to their ability to inhibit prokaryotic type II topoisomerases [4]. However, 4-quinolones can also exhibit non-classical bioactivities, including antitumor effects [5]. Voreloxin (**1**) (Figure 1), for instance, is a 4-quinolone analog that inhibits eukaryotic topoisomerase II and has achieved phase III clinical trials [6]. In previous work, our group synthesized a series of 4-quinolone-3-carboxamide derivatives, among which compounds **2a** and **2b** (Figure 1) presented significant cytotoxicity against gastric cancer cells ACP03, with IC_50_ values of 1.92 µM (1.39–2.66) and 5.18 µM (3.61–7.45), respectively (0.274 µM for doxorubicin). Both in vitro and in silico studies showed that these compounds are also able to inhibit topoisomerase II activity [7].

Other examples of 4-quinolone derivatives with anticancer profiles include natural alkaloids, such as compounds **3** and **4** (Figure 1). Derivative **3** exhibits activity against cervix adenocarcinoma (HeLa) and hepatocellular carcinoma (BEL7402) cell lines, with IC_50_ values of 18.53 ± 1.2 and 15.85 ± 1.4 µM, respectively (compared to IC_50_ values of 21.13 ± 1.9 and 19.3 ± 1.2 µM for doxorubicin) [8]. Compound **4**, known as intervenolin (Figure 1), demonstrates activity against human gastric cancer cells (MKN-74) with an IC_50_ value of 7.0 µM and against colorectal cancer cells (HCT-15) with an IC_50_ value of 3.7 µM [9,10]. The in vitro anticancer efficacy of intervenolin (**4**) was even higher when tested in these cancer cell lines cocultured with the corresponding stromal cells Hs738 (IC_50_ = 0.4 µM) and CCd-18Co (IC_50_ = 0.5 µM), suggesting enhanced effectiveness due to intercellular interactions resembling physiological conditions [11].

**Figure 2:**
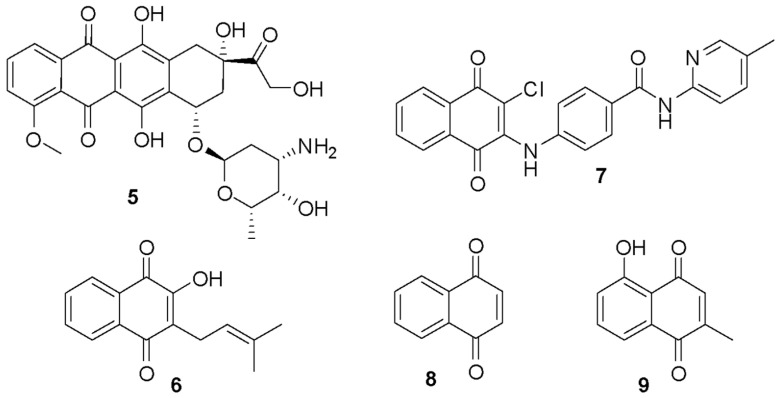
Doxorubicin (**5**) and representative examples of 1,4-naphthoquinone derivatives as anticancer agents (**6–9**).

Another important class of compounds recognized for their anticancer properties is 1,4-quinones [12,13,14]. Doxorubicin (**5**) (Figure 2), for instance, is an anthracycline 1,4-quinone derivative that has been used clinically since the 1960s in chemotherapy for various cancer types [15,16]. Lapachol (**6**) is a cytotoxic 1,4-naphthoquinone considered a potential anticancer drug that has reached clinical phase studies. However, these studies were discontinued due to an association with severe anemia, blood coagulation disorders, and gastrointestinal and renal toxicity [13,17]. Consequently, research into the preparation of new, more effective and selective antitumor compounds within this class has continued over the years. A recent example is compound **7**, which exhibits high cytotoxicity against MCF-7 human breast cancer cells [18].

Even structurally simple 1,4-quinone derivatives have significant cytotoxic potential in this context. 1,4-naphthoquinone itself (**8**) has a broad spectrum of antitumor activity, being able to potently inhibit angiogenesis and the growth of colon cancer cells (HCT116), for example, although in a non-selective way [19]. Another example is plumbagin (**9**), a natural compound isolated from *Plumbago zeylanica* L. that selectively induces apoptosis in refractory invasive prostate cancer cells (DU145) without affecting non-tumorigenic prostate epithelial cells (RWPE-1). Plumbagin also promoted tumor reduction by 90% in an in vivo screening after administration of 2 mg/kg (body weight) after three weeks of treatment [20].

The conjugation of two or more bioactive fragments within the same structure is a strategy used in medicinal chemistry for the rational design of new drug candidates. This approach aims to create compounds with a bioactive profile superior to that of the isolated original prototypes. This strategy can be particularly valuable for developing compounds with multitarget action that are capable of interacting with different receptors or through different mechanisms [21,22].

**Figure 3:**
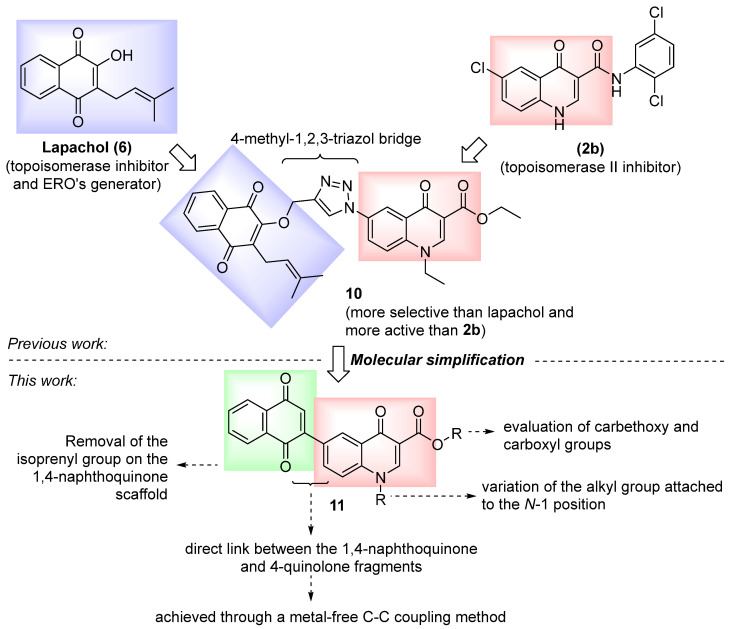
Our previous work described prototype **10**. Here, we show the rational design of the new 1,4-naphthoquinone/4-quinolone conjugates (**11**) with anticancer profile.

Recently, we synthesized a series of lapachol/1,2,3-triazol/4-quinolone hybrids based on this strategy. Among the conjugates obtained, compound **10** (Figure 3) demonstrated activity against three different breast cancer cell lines: MCF-7, MDA-MB-231, and 4T1. This compound exhibited high selectivity, showing no significant cytotoxicity against the non-cancerous cell line MCF10A, unlike doxorubicin [23]. Moreover, in the MCF-7 cell line, compound **10** selectively promoted apoptosis, decreased intracellular ATP levels without altering mitochondrial potential, increased reactive oxygen species production, and reduced cell glucose consumption and lactate production, which are critical in the central metabolic pathway for cancer cells [23]. Biological properties have been identified by our research group, confirming the success of the conjugation strategy between these two frameworks of medicinal chemistry interest in identifying new bioactive prototypes [24,25]. 

Considering the need to develop new antitumor agents with enhanced efficiency and selectivity, as well as the recognized antitumor activity associated with 1,4-naphthoquinone and 4-quinolone derivatives, and the success of our group in identifying bioactive substances through the molecular hybridization between these two nuclei, this work explores the preparation and anticancer potential of a new kind of 1,4-naphthoquinone/4-quinolone conjugates (**11**). These compounds are designed from the molecular simplification of hybrid **10** by modifying the bridge between the two main structural fragments with a direct C-C link in order to evaluate the effect of the direct conjugation on the antitumor activity (Figure 3).

## 2. Results and Discussion

### 2.1. Chemical Synthesis

**Scheme 1:**
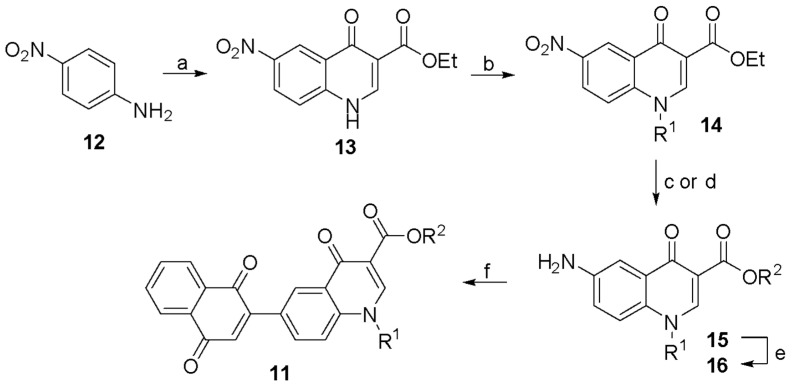
Synthetic route towards 1,4-naphthoquinone/4-quinolone conjugates. a: (1) EMME, EtOH; (2) Diphenylether, 250 °C; b: (1) K_2_CO_3_, DMF, r. t., 15 min. (2) R1-X, DMF, 80 °C, 24 h.; c: H_2_ (20 bar), Pd/C (10%), EtOH, 50 °C, 3 h. (for R1 = ethyl and pentyl); d: NH_4_Cl 0,05 M, Fe, reflux (for R1 = benzyl); e: (1) NaOH, EtOH, r. t. (2) H_2_O, HCl; f: t-butyl nitrite, DMSO, H_2_O, 1,4-naphthoquinone, ultrasound, 25 °C. (**15a/11a**: R^1^ = Et, R^2^ = Et; **15b/11b**: R^1^ = Bn, R^2^ = Et; **15c**/**11c**: R^1^ = Pentyl, R^2^ = Et; **16a**/**11d**: R^1^ = Et, R^2^ = H; **16b**/**11e**: R^1^ = CH_2_C_6_H_5_, R^2^ = H; **16c**/**11f**: R^1^ = C_5_H_11_, R^2^ = H).

Initially, 4-nitroaniline (**12**) was reacted with diethyl ethoxymethylenemalonate (EMME) followed by thermocyclization by treatment with refluxing diphenylether as a solvent, yielding nitro-4-quinolone intermediate **13** [26,27]. Compound **13** was subjected to an *N*-alkylation step through treatment with potassium carbonate, followed by a reaction with different alkyl halides. Subsequently, the 1-alkyl-nitro-4-quinolone intermediates (**14**) were chemically reduced. Derivatives **14a** and **14b** were converted into their amino counterparts (**15a** and **15b**) using hydrogenation in the presence of palladium adsorbed on carbon [25,28], while the benzylated derivative **14c** was treated with powdered iron as a reducing agent in the presence of an aqueous solution of ammonium chloride under reflux [27,28]. The amino esters (**15**) were subsequently hydrolyzed to the respective amino acids (**16**) by treatment with sodium hydroxide in an ethanolic solution, followed by the careful addition of aqueous hydrochloric acid until a pH of 6–7 was reached [28]. The 1-alkyl-amino-4-quinolones (**15** and **16**) were then submitted to the C-C cross-coupling step using the methodology described by Lamblin et al. [29]. The strategy involves the use of tert-butyl nitrite as a diazotizing agent without the need for metal catalysts or promoters. The method had to be adapted to the substrates used in this work, and the reaction took place in a 1:1 water/dimethyl sulfoxide system as a solvent, using ultrasound as an energy source (Scheme 1).

The structures of the new compounds **11a**–**f** were confirmed by ^1^H and ^13^C Nuclear Magnetic Resonance (^13^C NMR) spectroscopy and by high-resolution mass spectrometry analysis (HRMS), as discussed below.

### 2.2. Structure Characterization

The success of the C-C coupling reactions was confirmed by the ^1^H NMR, ^13^C-APT NMR, and HRMS spectra analyses. Taking conjugate **11a** as a representative example, two characteristic singlets, at 8.72 and 7.24 ppm, were readily identified in the ^1^H NMR spectrum and were assigned to the H-2 and H-3′ resonances. H-2 was the most unshielded among all the hydrogens present in the structure, which can be justified by the electron-withdrawing effects associated with the two conjugated carbonyls in C-4 and CO_2_Et and by the nitrogen in N-1. The olefin-type hydrogen, H-3′, is also unshielded by the mesomeric effect exerted by the carbonyl on C-1′, which justifies its low field resonance when compared to conventional olefin hydrogens. H-5 could also be easily associated with the doublet at 8.50 ppm, due to its coupling constant value consistent with the ^1^H X ^1^H meta coupling with H-7 (*J* = 2.1 Hz). Analysis of the homonuclear two-dimensional correlation spectrum, ^1^H X ^1^H–COSY, allowed the assignment of the double doublet at 8.02 ppm (*J* = 8.9 and 2.1 Hz) to H-7. This signal could be distinguished due to its characteristic multiplicity, although it had been partially superimposed on the multiplet at 8.06–8.02 ppm. Besides this multiplet, another one was identified at 8.14–8.11 ppm, having both shown electronic integration relative to one hydrogen each. Although their unambiguous differentiation was not possible, these signals were attributed to the resonances of H-5′ and H-8′. A set of superimposed signals in the range of 7.94–7.90 ppm, with total electronic integration relative to three hydrogens, was associated with H-8, H-6′, and H-7′, which was also confirmed due to the COSY correlations observed to the signals referring to H-5′/8′ and H-7. As for the hydrogens present in the aliphatic groups, two quartets at 4.45 (*J* = 7.2 Hz) and 4.25 (*J* = 7.2 Hz) ppm and two triplets at 1.41 (*J* = 7.2 Hz) and 1.31 (*J* = 7.2 Hz) ppm were assigned to the methylene and methyl hydrogens of the N-ethyl (NCH_2_CH_3_) and carbethoxyl (CO_2_CH_2_CH_3_) groups, respectively.

**Figure 4:**
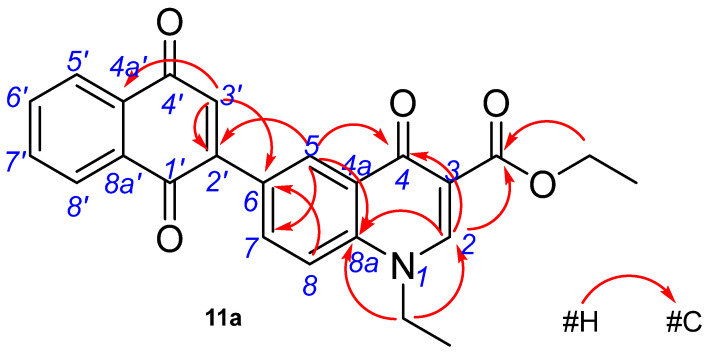
Main HMBC correlations observed for derivative **11a**.

To interpret the signals observed in the ^13^C-APT NMR spectrum, it was necessary to analyze the two-dimensional heteronuclear correlation spectra, ^1^H X ^13^C-HSQC (^1^*J*_CH_) and HMBC (^n^*J*_CH_, n = 2 or 3), in parallel. From the one bond correlation (^1^*J*_CH_) observed in the HSQC spectrum, it was possible to unambiguously assign the signals at 149.1, 135.0, 133.5, 128.1, 59.9, 48.0, 14.3, and 14.3 ppm to C-2, C-3′, C-7, C-5, CO_2_CH_2_CH_3_, NCH_2_CH_3,_ and the two methyl carbons (CO_2_CH_2_CH_3_, NCH_2_CH_3_), respectively. The resonance at 117.1 ppm was assigned to C-8. This signal presented an HSQC correlation with the superimposed ^1^H NMR signals at 7.94–7.90 ppm. Complementarily, its assignment was also made based on previously described NMR data for analogous structures. Although it was not possible to differentiate them, the signals at 126.5 and 125.5 ppm were assigned each to either C-5′ or C-8′, due to the HSQC correlation with H-5′ and H-8′. Likewise, C-6′ and C-7′ had their resonance attributed to signals at 134.1 or 134.2 ppm, respectively. Even with the analysis of the HMBC heteronuclear correlation spectrum (^n^*J*_CH_, n = 2 or 3), unambiguous differentiation of these signals was not possible. C-3 could be identified at 110.8 ppm; this chemical shift value characteristic of this carbon in 4-quinolone derivatives is shielded in opposition to other aromatic carbons due to the mesomeric electron-donating effect exerted by N-1. The signal at 129.6 ppm was assigned to C-6 due to the correlations (^3^*J*_CH_) observed with H-8 and H-3′ in the HMBC spectrum. C-2′ had its resonance identified at 146.2 ppm, due to the correlations observed with H-3′ (^2^*J*_CH_) and H-5 (^3^*J*_CH_). The three bond correlations observed with H-2, H-7, H-5, and NCH_2_CH_3_ allowed the attribution of the signal at 139.4 ppm to the C-8a. C-4a′ was associated with the signal at 131.7 ppm due to the correlation observed with H-3′ (^3^*J*_CH_). The two signals at 132.1 and 127.8 ppm, each associated with C4a or C-8a′, respectively, could not be unequivocally differentiated. Finally, a set of four low-field signals referring to the four carbonyl carbons of the structure were identified at 164.4, 172.7, 183.8, and 184.5 ppm. CO_2_CH_2_CH_3_ and C-4 could be unequivocally associated with the signals at 164.4 and 172.7 ppm, due to the heteronuclear correlations (^3^*J*_CH_) observed with CO_2_CH_2_CH_3_ and H-5, respectively. Both CO_2_CH_2_CH_3_ and C-4 had a common three-bond correlation with H-2. The quinone carbonyls, C-1′ and C-4′, could not be differentiated from each other, being associated with signals at 183.8 and 184.5 ppm. Figure 4 illustrates the main HMBC correlations observed for compound **11a**.

Considering the specificities inherent to each structure, the other conjugates **11b**–**f** presented ^1^H and ^13^C NMR signal patterns similar to those described for the derivative 11a. Furthermore, all substances had their molecular formulas confirmed through High-Resolution Mass Spectrometry (HRMS) analysis. These data are described in Section 3, and ^1^H and ^13^C-APT NMR spectra are available in the Supplementary Materials File (Figures S2–S38).

### 2.3. Antitumor Effects

#### 2.3.1. Cytotoxicity

Our first approach to assessing the anticancer activity of the novel 1,4-naphthoquinone/4-quinolone conjugates **11a**–**f** on breast cancer cells involved comparing their effects in the MTT cell viability assay to determine the half-maximal inhibitory concentration (IC_50_) in the 2D cell culture model. Doxorubicin and 1,4-naphthoquinone were used as controls. Concentration-response curves were obtained with the conjugates at concentrations ranging from 0 to 200 µM. According to the IC_50_ values obtained (Table 1), compounds **11a** and **11b** showed higher potency than 1,4-naphthoquinone in both MCF-7 and MDA-MD-231 cell lines. Moreover, these derivatives demonstrated similar potency to doxorubicin in MCF-7 cells. Compound **11e** demonstrated anticancer activity in both MCF-7 and MDA-MD-231 cell lines, comparable to the most active derivatives at low concentrations (<25 µM). However, its antitumor effect appeared to plateau at concentrations higher than 25 µM (Figure S1, Supplementary Materials), likely due to its low solubility in the medium, which hindered the determination of its IC_50_ value. This observation aligns with the structural characteristics of this derivative, which features the largest alkyl substituent at position R^1^. Finally, none of the molecules showed greater potency than doxorubicin for MDA-MB-231.

#### 2.3.2. Selectivity Indices (SI)

To increase the comprehension of the antitumor effects of our conjugates, an additional set of cell viability curves was performed with the breast cell line MCF10A, and their IC_50_ values were calculated and shown in Table 1. The IC_50_ values for these non-tumor cells ranged from 1.49 ± 1.43 for doxorubicin to 21.49 ± 1.2 for compound **11d**. The ratio of the IC_50_ values of the non-tumor and tumor cell lines was used to obtain a selectivity index for these compounds. The best results are those with values greater than 1, which correspond to compounds that are more toxic to tumor cells than to non-tumor cells [30]. Doxorubicin and 1,4-naphthoquinone were used for comparison, and both had very low SI values, indicating high toxicity to non-tumor cells. The SI values calculated for compounds **11a** and **11b** show their relative cytotoxicity towards tumor cells compared to non-tumor MCF10A cells (Figure S1). The SI values for these derivatives suggest a more selective profile comparable to 1,4-naphthoquinone and doxorubicin. Compound **11a**, for instance, presents potency similar to doxorubicin in the MCF-7 cell line, but with a cytotoxicity effect in MCF10A seven times lower. In general, SI values for all the synthesized derivatives (except for **11f**) were superior to 1,4-naphthoquinone and doxorubicin for both tumor cell lines, including SI values higher than 1, which indicate lower toxicity, making them good drug candidates for further investigation.

#### 2.3.3. Apoptosis Induction

**Figure 5:**
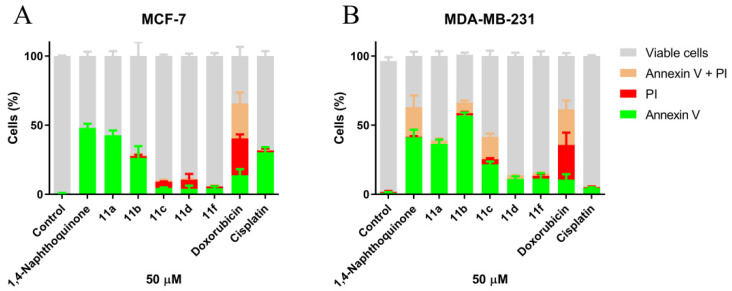
Naphthoquinone-quinolone hybrids induce apoptosis in MCF-7 (**A**) and MDA-MB-231 (**B**) cells at different levels. Cells were treated with compounds at 50 µM and exhibited markers of early and late apoptosis (n = 3).

We investigated apoptosis induction through the annexin V/PI protocol, as described in Section 3. Doxorubicin, 1,4-naphthoquinone, and cisplatin were used as controls. For MCF-7 cells, as can be seen in Figure 5A, at 50 µM, early apoptosis induction was comparable for 1,4-naphthoquinone, compound **11a**, **11b**, and cisplatin. Doxorubicin, on the other hand, which has been described to act through different mechanisms to induce cell death [31], shows early apoptosis but also shows late apoptosis/necrosis in a great proportion. Compounds **11c**–**f**, at the concentration and time used here, depicted a very low degree of early apoptosis induction, indicating that the cell viability reduction observed in the MTT assays might occur through other mechanisms. For MDA-MB-231 cells (Figure 5B), the effects observed were more prominent at the concentrations tested. All compounds, except for **11d** and **11f,** displayed similar apoptosis profiles to 1,4-naphthoquinone. Doxorubicin had effects similar to those seen on MCF-7 cells, and cisplatin had a lower effect on MDA-MB-231 cells.

#### 2.3.4. Clonogenic Assay

**Figure 6:**
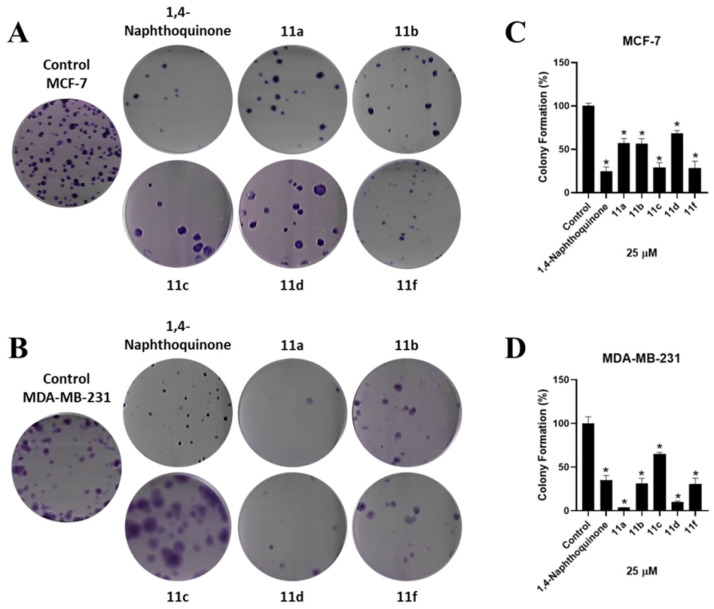
The inhibition of colony formation by compounds **11a–f** using MCF-7 (**A**,**C**) and MDA-MB-231 (**B**,**D**) cell lines. Cells were treated with derivatives **11a–f** at 25 µM for 24 h. Subsequently, 400 cells were plated on 6-well plates for approximately 14 days. Then, cells were stained with crystal violet dye to visualize the colonies formed. Significant reductions in colony formation were observed in the presence of all compounds and 1,4-naphthoquinone, compared to the control. * *p* < 0.05, Student’s *t*-test, n = 3.

The clonogenic assay is an effective tool for investigating individual cells’ ability to form colonies. Tumor cells with the ability to generate an expanding family of descendants are clonogenic. Therefore, a clonogenic assay is a simple method to verify one of the important characteristics of cancer cells, which is the ability to detach from the site of origin and to form metastases in other locations [32]. Empirical evidence indicates that only a minority of cells within a tumor exhibit clonogenic properties, with the majority being non-clonogenic and dying without any therapy after a few cell divisions. For instance, 90–99% of the tumor cells in FaDU tumors, a type of human squamous cell carcinoma observed in nude mice, are non-clonogenic [33]. To achieve the main goal of cancer treatment, which is tumor eradication, it is necessary to inactivate all clonogenic cells through either cell killing or the induction of a permanent state of dormancy, thereby eliminating their clonogenic capacity. The assessment of the potential of a drug to achieve a curative outcome, i.e., effectively neutralizing clonogenic tumor cells, requires the utilization of experimental endpoints that accurately reflect the response of clonogenic cells. Clonogenic endpoints hold particular significance when novel anticancer agents are incorporated into curative therapeutic regimens, such as in combination with radiotherapy or chemotherapy [34]. Thus, clonogenic assays were conducted for compounds **11a**–**f** and 1,4-naphthoquinone. Figure 6 shows the decrease in colony formation with cells subjected to a 24-h treatment with the compounds. This effect was observed in both cell lines: all compounds at 25 µM significantly reduced colony formation, with the most effective being **11c** and **11f** for MCF-7 (Figure 6A,C) and **11a** and **11d** for MDA-MB-231 cells (Figure 6B,D), evidencing their potential to eliminate the clonogenic potential of these cell lines.

#### 2.3.5. Duration and Reversibility of the Effects

**Figure 7:**
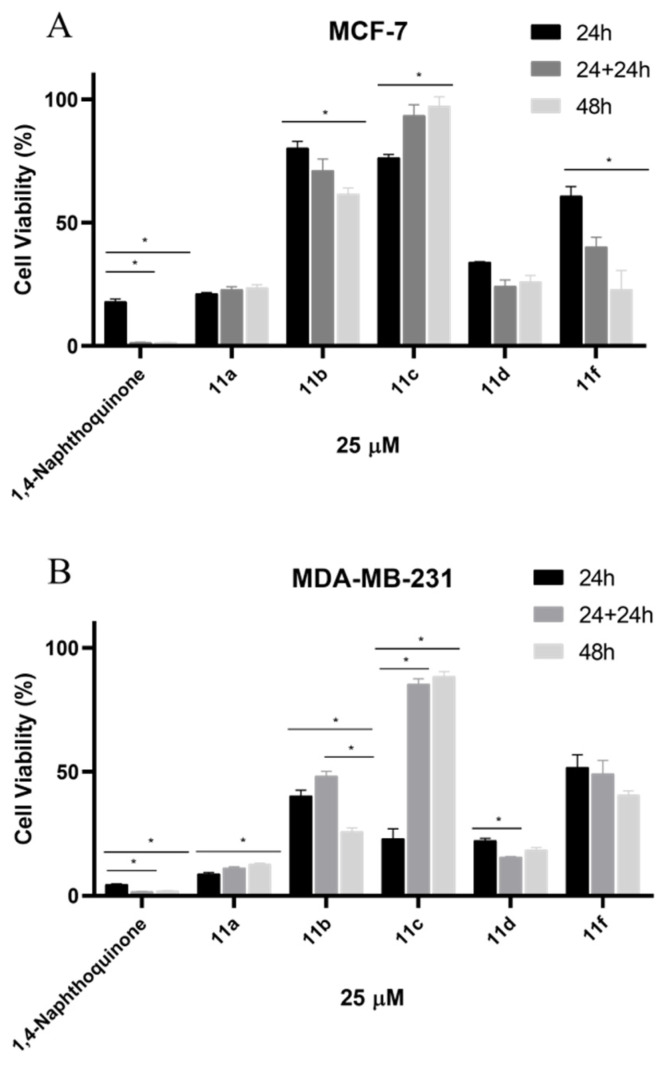
Reversibility assay: investigating the effects of treatment durability on cell viability of (**A**) MCF-7 cells and (**B**) MDA-MB-231 cells. Cells were treated in three different manners: (1) for 24 h, continuously; (2) for 24 h, followed by removal of the treatment and addition of fresh medium, then further cultivation for 24 h; and (3) for a total period of 48 h. Cell viability was measured using the MTT assay. * *p* < 0.05, Student’s *t*-test, n = 3.

Our next issue concerned the reversibility of the effects: whether they persisted through a longer time of treatment, or if they ceased after the removal of the treatments. To investigate this, we used each compound at 25 µM and treated the MCF-7 and MDA-MB-231 cells for 24 h and 48 h. The 48-h treatment group was further subdivided into two different categories: the first was treated for 24 h, after which the culture medium was replaced with fresh medium without the compound, and then incubated for an additional 24-h period (24 + 24 h). In the second category, cells were continuously exposed to the compound for the entire 48-h duration, without any handling during this period. Cell viability was assessed as the evaluation parameter. The results are shown in Figure 7A (MCF-7) and Figure 7B (MDA-MB-231). Except for **11c**, which displays a short effect (the effect seen with 24 h does not withstand until 48 h, either with the maintenance of the treatment or not), all compounds appear to have an irreversible effect at 25 µM, including 1,4-naphthoquinone. In this assay, 11c is not capable of sustaining the antitumor effect after 48 h of treatment, with or without its removal (Figure 7B). On the other hand, this compound induces apoptosis in the same cell line (Figure 5B). Also, while it promotes a reduction in the number of colonies formed (Figure 6D), Figure 6B shows that they are larger than those of the control, which indicates an increase in cellular proliferation of surviving cells. In MCF-7 cells, the effect observed is similar, with a lower intensity of apoptosis induction. These results are indicative of different cellular mechanisms of apoptosis induction and cell survival triggered by our compounds in the two cell lines tested.

#### 2.3.6. 3D-Cell Culture Viability

**Figure 8:**
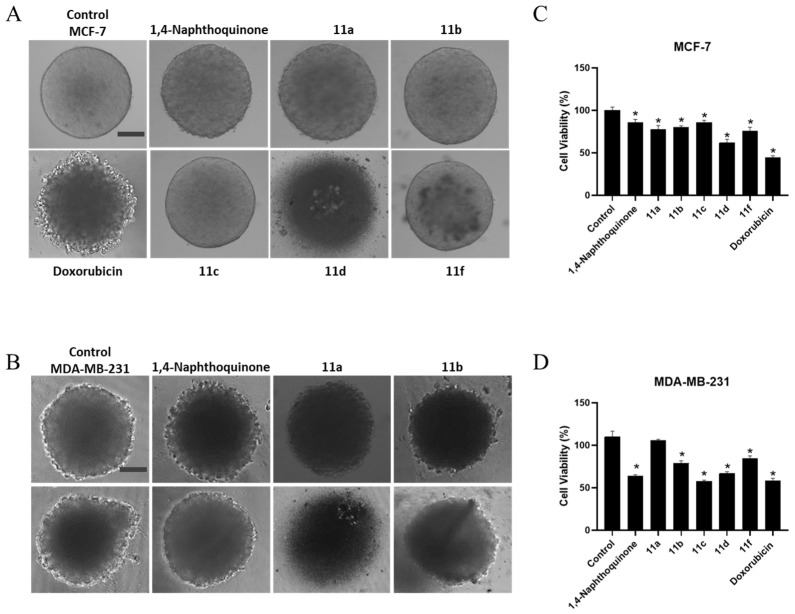
Compounds **11a–f** reduce breast cancer cell viability in a 3D cell culture model. Spheroids from MCF-7 cells (**A**) and MDA-MB-231 cells (**B**) were prepared and then treated with compounds at 25 µM for 24 h. Spheroids were assessed for viability using the acid phosphatase method (**C**,**D**) and a significant decrease was observed for all compounds, except for **11a** in MDA-MB-231 cells. 1,4-naphthoquinone and doxorubicin were used as controls. Scale bar, 100 µm. * *p* < 0.05, Student’s *t*-test (n = 3).

3D-cell cultures display features that resemble in vivo tumors: due to their tridimensional structure, cells are not provided with the same amounts of oxygen and nutrients in the center as on the surface, resulting in a gradient that leads to the formation of a necrotic core, similar to the in vivo tumor environment [35]. In the 3D-cell culture model used here, spheroids are formed due to low interaction with the microplate bottom, promoted by its coating with agarose [36], and strong cell-to-cell interactions in MCF-7 cells, generating a finely compact and homogeneous spheroid. For MDA-MB-231 cells, the addition of collagen I is an extra stimulus necessary for spheroid stabilization [37]. Spheroids were prepared and grown for 48 h and then treated with the compounds at 25 µM. After 48 h, morphological changes were observed, and their viabilities were measured as described in Section 3. The exposure to the compounds for 48 h promoted visible changes in the morphologies of spheroids for both cell lines when compared to the controls (Figure 8). We observed changes in the morphology of MCF-7 spheroids treated with 1,4-naphthoquinone, **11a**, **11d**, and doxorubicin (Figure 8A). While it is more complicated to imply changes in the proliferative (surface) region of the MDA-MB-231 spheroids (Figure 8B), they are observed together with morphological changes after treatment, making them appear more crumbly and lose their round shape. These findings are in line with the viability test, which detected a reduction in the viabilities for all of them, which is significant compared to the control (*p* < 0.05), except for 11a in MDA-MB-231 spheroids (Figure 8C,D). We should also highlight that the concentrations necessary for an effect on spheroids are expected to be higher when compared to 2D-cell culture models [38]. Here, we observed changes using 25 µM of each compound, as performed in the other experiments.

#### 2.3.7. In Silico Drug-Likeness and ADME

A thorough analysis of the physicochemical properties of the compounds **11a**–**f** is detailed in Table 2, utilizing the Swiss ADME server [39]. These properties are crucial indicators of pharmacokinetics and the potential of a molecule as a drug. Typically, orally active drugs have molecular weights between 160 and 480 Da [40]. According to Table 2, all the naphthoquinone-quinolone hybrids investigated here fall within this range.

Molecular flexibility, determined by the number of rotatable bonds, significantly affects the bioavailability of a molecule. Compounds with fewer than ten rotatable bonds, as observed in Table 2 for compounds **11a**–**f**, generally exhibit higher oral bioavailability. Molecular refractivity (MR) provides insight into the size and electronic properties of a molecule, and it has been correlated with drug permeability. The qualifying range for the MR of drug-like compounds is between 40 and 130 [39], and all compounds **11a**–**f** meet this criterion. Total polar surface area (TPSA) reflects the ability of a molecule to interact with biological membranes. Compounds exhibiting TPSA values less than 60Å^2^, such as those found for compounds **11a**–**f**, indicate good drug absorption in the intestine. Furthermore, MlogP, an indicator of lipophilicity, is essential as it significantly impacts the pharmacokinetic properties of compounds. Poor absorption or permeation is more likely when MlogP is greater than 4.15 [41]. As shown in Table 2, all compounds have MlogP values below 2.7. ESOL predicts the water solubility of these compounds, and all hybrids are categorized as moderately soluble (MS). Additionally, compounds **11a**, **11b**, **11d**, **11e**, and **11f** exhibited zero violations of the drug-likeness rules of Lipinski, Ghose, Veber, Egan, and Muegge. Compound **11c** presented only one violation of the Muegge (XLogP3 > 5) rule. Overall, the results indicate that compounds **11a**–**f** are good orally active drug candidates.

## 3. Materials and Methods

### 3.1. Synthesis

All reagents and solvents were purchased from Merck KGaA (Darmstadt, Germany) and used without further purification. Melting points were measured with a Fisher–Johns apparatus. NMR spectra were recorded on a Varian spectrometer operating at 500.00 MHz (^1^H) and 125.00 MHz (^13^C) or at 300.00 MHz (^1^H) and 75.0 MHz (^13^C) using DMSO-*d*_6_ as the solvent. Chemical shifts were reported in parts per million (ppm) relative to the internal standard tetramethylsilane (TMS). Hydrogen and carbon NMR spectra were typically obtained at room temperature. The two-dimensional experiments were conducted using standard Varian Associates automated programs used for data acquisition and processing. Compounds **13**, **14a**–**c**, **15a**–**c**, and **16a**–**c** were synthesized as described in previous works [25,42,43].

C-C coupling. General procedure for the synthesis of 1,4-naphthoquinone/4-quinolone conjugates (**11a**–**c**).

1,4- naphthoquinone (8) (0.85 mmol, 0.135 g) was dissolved in 6.0 mL of a 1:1 mixture of DMSO and water. To this solution, *tert*-butyl nitrite (1.0 mmol, 0.13 mL) was added, and then the respective 6-amino-4-quinolone **15a**–**c** or **16a**–**c** (0.77 mmol). The reaction medium was sonicated at room temperature for 90 min until the gas evolution ceased. The precipitated solid was filtered, washed with distilled water, and dried under a vacuum. The products were purified by crystallization in dichloromethane/petroleum ether 1:1 or by preparative Thin Layer Chromatography using dichloromethane/methanol 15:1 as eluent.

Ethyl 6-(1,4-dioxo-1,4-dihydronaphthalen-2-yl)-1-ethyl-4-oxo-1,4-dihydroquinoline-3-carboxylate (**11a**): 62% yield; orange solid; m. p. 220–222 °C. IR (ν): 2987 (C-H), 1721 (C=O), 1667 (C=O), 1651 (C=O) and 1627 (C=C) cm^−1^. ^1^H NMR (300.00 MHz, DMSO-*d*_6_) δ: 8.72 (s, 1H, H-2), 8.50 (d, 1H, *J* = 2.1 Hz, H-5), 8.14–8.11 (m, 1H, H-5′ or H-8′), 8.06–8.02 (m, 1H, H-5′ or H-8′), 8.02 (dd, 1H, *J* = 8.9 and 2.1 Hz, H-7), 7.94–7.90 (m, 3H, H-8, H-6′ and H-7′), 7.24 (s, 1H, H-3′), 4.45 (q, 2H, *J* = 7.2 Hz, NCH_2_CH_3_), 4.25 (q, 2H, *J* = 7.2 Hz, CO_2_CH_2_CH_3_), 1.41 (t, 3H, *J* = 7.2 Hz, NCH_2_CH_3_) and 1.31 (t, 3H, *J* = 7.2 Hz, CO_2_CH_2_CH_3_) ppm. ^13^C-APT NMR (75.0 MHz, DMSO-*d*_6_) δ: 184.5 (C-1′ or C-4′), 183.8 (C-1′ or C-4′), 172.7 (C-4), 164.4 (CO_2_CH_2_CH_3_), 149.1 (C-2), 146.2 (C-2′), 139.4 (C-8a), 135.0 (C-3′), 134.2 (C-6′ or C-7′), 134.1 (C-6′ or C-7′), 133.5 (C-7), 132.1 (C-4a or C-8a′), 131.7 (C-4a′), 129.6 (C-6), 128.1 (C-5), 127.8 (C-4a or C-8a′), 126.5 (C-5′ or C-8′), 125.5 (C-5′ or C-8′), 117.1 (C-8), 110.8 (C-3), 59.9 (CO_2_CH_2_CH_3_), 48.0 (NCH_2_CH_3_), 14.3 (NCH_2_CH_3_) and 14.3 (CO_2_CH_2_CH_3_) ppm. HRMS: *m*/*z* calcd for C_24_H_19_NO_5_ [M+H]+ 402.1341; found 402.1336.

Ethyl 1-benzyl-6-(1,4-dioxo-1,4-dihydronaphthalen-2-yl)-4-oxo-1,4-dihydroquinoline-3-carboxylate (**11b**): 57% yield; yellow solid; m. p. 191–193 °C. IR (ν): 2974 (C-H), 1721 (C=O), 1691 (C=O), 1666 (C=O) and 1647 (C=O) cm^−1^. ^1^H NMR (500.00 MHz, DMSO-*d*_6_) δ: 8.96 (s, 1H, H-2), 8.49 (d, 1H, *J* = 1.5 Hz, H-5), 8.13–8.08 (m, 1H, H-5′ or H-8′), 8.06–8.01 (m, 1H, H-5′ or H-8′), 7.95–7.88 (m, 3H, H-7, H-6′ and H-7′), 7.73 (d, 1H, *J* = 8.8 Hz, H-8), 7.22 (s, 1H, H-3′), 5.74 (s, 2H, NCH_2_), 4.26 (q, 2H, *J* = 7.0 Hz, CO_2_CH_2_CH_3_) and 1.31 (t, 3H, *J* = 7.0 Hz, CO_2_CH_2_CH_3_) ppm. ^13^C-APT NMR (125.00 MHz, DMSO-*d*_6_) δ: 184.41 (C-1′ or C-4′), 183.74 (C-1′ or C-4′), 172.74 (C-4), 164.32 (CO_2_CH_2_CH_3_), 150.01 (C-2), 146.05 (C-2′), 139.79 (C-8a), 135.70, 134.98 (C-3′), 134.07 (C-6′ or C-7′), 133.28, 132.08, 131.66, 129.90, 129.68, 128.88 (C-3″/5″), 127.96, 127.86, 127.83, 126.48, 126.39 (C-2″/6″), 125.37, 123.31, 118.51, 117.65, 111.06 (C-3), 59.85 (CO_2_CH_2_CH_3_), 55.62 (NCH_2_) and 14.18 (CO_2_CH_2_CH_3_) ppm. HRMS: *m*/*z* calcd for C_29_H_22_NO_5_ [M+H]^+^ 464.1492; found 464.1506.

Ethyl 6-(1,4-dioxo-1,4-dihydronaphthalen-2-yl)-4-oxo-1-pentyl-1,4-dihydroquinoline-3-carboxylate (**11c**): 74% yield, orange solid; m. p. 102–105 °C. IR (ν): 2957 (C-H), 1720 (C=O), 1664 (C=O) and 1607 (C=C) cm^−1^. ^1^H NMR (500.00 MHz, DMSO-*d*_6_) δ: 8.66 (s, 1H, H-2), 8.52 (d, 1H, *J* = 2.0 Hz, H-5), 8.15–8.12 (m, 1H, H-5′ or H-8′), 8.07–8.04 (m, 1H, H-5′ or H-8′), 8.02 (dd, 1H, *J* = 8.8 and 2.0 Hz, H-7), 7.95–7.90 (m, 2H, H-6′ and H-7′), 7.89 (d, 1H, *J* = 8.8 Hz, H-8), 7.23 (s; 1H, H-3′), 4.40 (t, 2H, *J* = 7.2 Hz, NCH_2_), 4.26 (q; 2H, *J* = 7.1 Hz, CO_2_CH_2_CH_3_), 1.82 (quint, 2H, *J* = 7.2 Hz, NCH_2_CH_2_), 1.40–1.33 (m, 4H, NCH_2_CH_2_(CH_2_)_2_) and 0.89 (t, 3H, *J* = 7.3 Hz, N(CH_2_)_4_CH_3_) ppm. ^13^C-APT NMR (125.00 MHz, DMSO-*d*_6_) δ: 184.20 (C-1′ or C-4′), 183.63 (C-1′ or C-4′), 172.36 (C-4), 164,23 (CO_2_CH_2_CH_3_), 149.04 (C-2), 146.03 (C-2′), 139.42 (C-8a), 134.79 (C-3′), 133.89 (C-6′ or C-7′), 133.86 (C-6′ or C-7′), 133.12 (C-7), 132.01 (C-4a′ or C-8a′), 131.58 (C-4a′ or C-8a′), 129.36 (C-6), 127.85 (C-5), 127.71 (C-4a), 126.31 (C-5′ or C-8′), 125.20 (C-5′ or C-8′), 116.92 (C-8), 110.64 (C-3), 59.53 (CO_2_CH_2_CH_3_), 52.51 (NCH_2_), 27.93 (NCH_2_CH_2_), 27.70 (NCH_2_CH_2_(CH_2_)_2_), 21.39 (NCH_2_CH_2_(CH_2_)_2_), 14.01 (CO_2_CH_2_CH_3_) and 13.44 (N(CH_2_)_4_CH_3_) ppm. HRMS: *m*/*z* calcd for C_27_H_25_NO_5_Na [M+Na]^+^ 466.1625; found 466.1625.

6-(1,4-dioxo-1,4-dihydronaphthalen-2-yl)-1-ethyl-4-oxo-1,4-dihydroquinoline-3-carboxylic acid (**11d**): 53% yield, brown solid; m. p. > 300 °C. IR (ν): 3364 (O-H), 2922 (C-H), 1725 (C=O), 1664 (C=O) and 1607 (C=C) cm^−1^. ^1^H NMR (500.00 MHz, DMSO-*d*_6_) δ: 14.97 (s, 1H, CO_2_H), 9.07 (s, 1H, H-2), 8.66 (d, 1H, *J* = 2.0 Hz), 8.18 (dd, 1H, *J* = 8.7 and 2.0 Hz, H-7), 8.16–8.11 (m, 1H, H-5′ or H-8′), 8.14 (d, 1H, *J* = 8.7 Hz, H-8), 8.10–8.05 (m, 1H, H-5′ or H-8′), 7.95–7.91 (m, 2H, H-6′ and H-7′), 7.31 (s, 1H, H-3′), 4.64 (q, 2H, *J* = 7.4 Hz, NCH_2_CH_3_) and 1.48 (t, 3H, *J* = 7.4 Hz, NCH_2_CH_3_) ppm. ^13^C-APT NMR (125.00 MHz, DMSO-*d*_6_) δ: 184.31 (C-1′ or C-4′), 183.52 (C-1′ or C-4′), 177.58 (C-4), 165.61 (CO_2_H), 149.37 (C-2), 145.56 (C-2′), 139.49 (C-8a), 135.39 (C-3′), 134.76 (C-7), 134.09 (C-6′ or C-7′), 134.03 (C-6′ or C-7′), 131.98 (C-4a, C-4a′ or C-8a′), 131.59 (C-4a, C-4a′ or C-8a′), 130.88 (C-6), 127.32 (C-5), 126.43 (C-5′ or C-8′), 125.33 (C-5′ or C-8′), 125.13 (C-4a, C-4a′ or C-8a′), 117.87 (C-8), 108.19 (C-3), 48.96 (NCH_2_CH_3_) and 14.39 (NCH_2_CH_3_). HRMS: *m*/*z* calcd for C_22_H_15_NO_5_Na [M+Na]^+^ 396.0842; found 396.0839.

1-benzyl-6-(1,4-dioxo-1,4-dihydronaphthalen-2-yl)-4-oxo-1,4-dihydroquinoline-3-carboxylic acid (**11e**): 49% yield, brown solid; m. p. > 300 °C. IR (ν): 2921 (C-H), 1721 (C=O), 1664 (C=O) and 1609 (C=C) cm^−1^. ^1^H NMR (500.00 MHz, DMSO-*d*_6_) δ: 9.26 (s, 1H, H-2), 8.66 (d, 1H, *J* = 2.7 Hz, H-5), 8.15–8.10 (m, 1H, H-5′ or H-8′), 8.07 (dd, 1H, *J* = 8.7 and 2.7 Hz, H-7), 8.07–8.03 (m, 1H, H-5′ or H-8′), 7.97 (d, 1H, *J* = 8.7 Hz, H-8), 7.94–7.88 (m, 2H, H-6′ and H-7′), 7.42–7.29 (m, 5H, H-2′/6′, H-3′5′ and H-4′), 7.28 (s, 1H, H-3′) and 5.91 (s, 2H, NCH_2_) ppm. ^13^C-APT NMR (125.00 MHz, DMSO-*d*_6_) δ: 184.14 (C-1′ or C-4′), 183.38 (C-1′ or C-4′), 177.72 (C-4), 165.35 (CO_2_H), 150.18 (C-2), 145.43 (C-2′), 139.87 (C-8a), 135.35 (C-3′), 134.96 (C-1″), 134.47 (C-7), 133.93 (C-6′ or C-7′), 133.89 (C-6′ or C-7′), 131.89 (C-4a or C-8a), 131.53 (C-4a or C-8a), 130.88 (C-6 or C-4a), 128.71 (C-3″/5″), 127.84 (C-4″), 127.17 (C-5) 126.47 (C-2″/6″), 126.29 (C-5′ or C-8′), 125.24 (C-6 or C-4a), 125.21 (C-5′ or C-8′), 118.23 (C-8), 108.38 (C-3) and 56.30 (NCH_2_) ppm. HRMS: *m*/*z* calcd for C_27_H_18_NO_5_ [M+H]^+^ 436.1179; found 436.1198.

6-(1,4-dioxo-1,4-dihydronaphthalen-2-yl)-4-oxo-1-pentyl-1,4-dihydroquinoline-3-carboxylic acid (**11f**): 57% yield, brown solid; m. p. > 300 °C. IR (ν): 2922 (C-H), 1720 (C=O), 1659 (C=O) and 1608 (C=C) cm^−1^. ^1^H NMR (500.00 MHz, DMSO-*d*_6_) δ: 9.05 (s, 1H, H-2), 8.65 (d, 1H, *J* = 1.7 Hz, H-5), 8.18 (dd, 1H, *J* = 8.7 and 1.7 Hz, H-7), 8.16–8.13 (m, 1H, H-5′ or H-8′), 8.12 (d, 1H, *J* = 8.7 Hz, H-8), 8.08–8.04 (m, 1H, H-5′ or H-8′), 7.94–7.92 (m, 2H, H-6′ and H-7′), 7.30 (s, 1H, H-3′), 4.59 (t, 2H, *J* = 7.3 Hz, NCH_2_), 1.86 (quint, 2H, *J* = 7.3 Hz, NCH_2_CH_2_), 1.38–1.35 (m, 4H, NCH_2_CH_2_(CH_2_)_2_) and 0.88 (t, 3H, J = 7.0 Hz, N(CH_2_)_4_CH_3_) ppm. ^13^C-APT NMR (125.00 MHz, DMSO-*d*_6_) δ: 184.20 (C-1′ or C-4′), 183.47 (C-1′ or C-4′), 177.51 (C-4), 165.50 (CO_2_H), 149.51 (C-2), 145.53 (C-2′), 139.64 (C-8a), 135.38 (C-3′), 134.62 (C-7), 133.99 (C-6′ or C-7′), 133.96 (C-6′ or C-7′), 131.95, 131.57, 130.83 (C-6), 127.22 (C-5), 126.35 (C-5′ or C-8′), 125.27 (C-5′ or C-8′), 125.12, 117.85 (C-8), 108.01 (C-3), 53.55 (NCH_2_), 28.17 (NCH_2_CH_2_), 27.67 (NCH_2_CH_2_(CH_2_)_2_), 21.41 (NCH_2_CH_2_(CH_2_)_2_) and 13.45 (N(CH_2_)_4_CH_3_) ppm. HRMS: *m*/*z* calcd for C_25_H_21_NO_5_Na [M+Na]^+^ 438.1312; found 438.1306.

### 3.2. Biological Assays

#### 3.2.1. Cell Lines

Cell lines were obtained from the Rio de Janeiro Cell Bank. MDA-MB-231 and MCF-7 cells were maintained in DMEM supplemented with 10% fetal bovine serum and 10 μg/mL gentamicin (Gibco Scientific, Grand Island, NY, USA). MCF10A cells were maintained in DMEM supplemented with 10% fetal bovine serum, Mammary Epithelial Growth Supplement (Gibco Scientific, Grand Island, NY, USA), and 10 μg/mL gentamicin (Gibco Scientific, Grand Island, NY, USA). All cell lines were maintained at 37 °C with 5% CO_2_.

#### 3.2.2. MTT Assay

Cells were plated in 96-well plates to a confluence of 80–90% after 24 h. Then, the cell medium was removed and 100 μL of medium containing different concentrations of the studied compounds (0.1% DMSO) were added to the wells that were incubated for 24 h. Later, the cell medium was removed, and 0.5 mg/mL MTT in PBS was added to the wells, and cells were kept at 37 °C with 5% CO_2_ for 2 to 4 h. DMSO (100 μL) was added to solubilize the formazan crystals, and the plate was analyzed at 570 nm and 650 nm [44] in a SpectraMax Paradigm Multi-Mode Microplate Reader (Molecular Devices, San Jose, CA, USA).

#### 3.2.3. Clonogenic Assay

Cells were plated in 24-well plates to a confluence of 70% to 80% after 24 h. Then, the cell medium was removed, and the fresh medium containing the compounds was incubated for 24 h. The control wells contained 0.1% DMSO, equivalent to the DMSO concentration in the treated wells. After the treatment, cells were trypsinized, and 400 viable cells (counted with Trypan blue) were plated in 6-well plates and incubated until visible colony formation in the control wells for approximately 14 days. On the final day, the medium was removed, and the wells were dyed with a crystal violet solution (2.5 mg/mL in 20% methanol) for 30 min and washed with distilled water. The plates were digitally imaged, and the colonies formed were counted manually [45].

#### 3.2.4. Apoptosis Assay

For the apoptosis assay, the manufacturer’s instructions were followed (Dead Cell Apoptosis Kit with Annexin V for Flow Cytometry, Thermo Fisher Scientific, MA, USA). The cells were analyzed in a Countess II FL cell counter (Thermo Fisher Scientific, Waltham, MA, USA).

#### 3.2.5. 3D-Cell Culture

The procedure was performed as described by Gong et al., with a few modifications [36]. A total of 5000 cells per well were plated on 96-well plates with bottoms previously covered with 50 µL of a 1% agarose solution. The plates were then centrifuged at 300× *g* for 10 min. For MDA-MB-231 cells, collagen I from rat tail (Gibco) was added to a 3 µg/mL final concentration in the cell suspension. The plates were incubated at 37 °C with 5% CO_2_ for 48 h. The cell spheroids/aggregates were treated with fresh medium added to the compounds and their viability was evaluated after 48 h. Spheroids were photographed with an inverted microscope Bel INV-100 (Bel Engineering, Monza, Italy) equipped with a digital camera.

#### 3.2.6. Acid Phosphatase Assay

To measure spheroids viability, this viability assay was performed following Friedrich et al. [46]. The medium was removed from wells and spheroids were washed with PBS. Then, 100 µL of a solution containing 2 mg/mL p-nitrophenyl phosphate (PNPP), 0.1 M citric acid, and 0.1% (*v*/*v*) Triton X, pH 5.0 were added. After two hours at 37 °C, 10 µL of 1M NaOH was applied to each well and after 10 min the absorbance was assessed at 405 nm in SpectraMax Paradigm Microplate Reader (Molecular Devices, San Jose, CA, USA).

#### 3.2.7. In Silico Drug-Likeness and ADME

The SwissADME online server [39] (accessed on 16 May 2024) was utilized to investigate the in silico pharmacokinetic features of the naphthoquinone-quinolone derivatives, including lipophilicity, gastrointestinal absorption, and drug-likeness. Additionally, the physicochemical properties, such as topological polar surface area, number of hydrogen bond donors, number of hydrogen bond acceptors, and number of rotatable bonds, were predicted.

#### 3.2.8. Statistical Analyses, IC_50_, and Selectivity Index Calculation

Statistical analyses were performed using SigmaPlot for Windows version 12 (Systat Software, Inc, San Jose, CA, USA). The results were expressed as means and standard error of triplicate determinations and analyzed by Student’s *t*-test. The IC_50_ values were calculated using GraphPad Prism v. 8.0.1, and the selectivity index was estimated as the ratio between the IC_50_ value of MCF10A/IC_50_ value of MDA-MB-231 or MCF-7 [33], for each compound.

## 4. Conclusions

In this work, we aimed to evaluate the effects of naphthoquinone-quinolone derivatives on breast cancer cell lines. The cells used in this study, MCF-7 (luminal) and MDA-MB-231 (triple-negative), are representative of the breast cancer types for which new choices for therapy are still urgent. MDA-MB-231 and MCF-7 cells exhibit different features that correlate with their molecular classification, such as the expression of hormonal receptors [47]. Also, as expected for a TNBC cell line, MDA-MB-231 cells are more aggressive, with a mesenchymal-like pattern in which cells lose their cell-to-cell interactions, becoming more invasive and with higher motility [48]. The molecular pathways activated by drugs vary between the two cell lines due to different mutations. For instance, MDA-MB-231 has a p53 mutation [49], which makes it more resistant to DNA-damaging drugs such as cisplatin. The findings from our study underscore the promising potential of the investigated compounds as effective anticancer agents. Notably, compounds **11a** and **11b** were found to induce apoptosis, a crucial mechanism for inhibiting cancer cell proliferation and promoting cell death, while the remaining compounds appeared to exert their effects through alternative mechanisms. Furthermore, all compounds significantly reduced colony formation, indicative of their ability to impair the clonogenic potential of MCF-7 and MDA-MB-231 cells. Specifically, compounds **11c** and **11f** demonstrated greater efficacy against MCF-7 cells, while compounds **11a** and **11d** were more effective against MDA-MB-231 cells, highlighting their differential effects on distinct cancer cell lines. Moreover, our experiments revealed that the effects of all compounds, except for **11c**, were irreversible, suggesting sustained and potent anticancer activity. Additionally, the 3D-cell culture assays, which better mimic in vivo tumor environments, except for **11a,** corroborated the results observed in the viability studies, further emphasizing the promising therapeutic prospects of these compounds. ADME feature predictions confirmed the promising characteristic of compounds **11a**–**f** in terms of pharmacokinetics properties. Together, these findings provide compelling evidence for the potential of the investigated compounds as effective and selective anticancer agents. Further studies, including the investigation of their mechanisms of action and also pre-clinical studies to address their safety and in vivo effects would be useful to define their clinical utility.

## Figures and Tables

**Table 1:** IC_50_ values and selectivity indices (SI) of 1,4-naphthoquinone, compounds **11a–f**, and doxorubicin.

	IC_50_ ± SEM (µM)			SI	
Compound	MCF-7	MDA-MB-231	MCF10A	IC_50_ MCF10A/IC_50_ MCF-7	IC_50_ MCF10A/ IC_50_ MDA-MB-231
1,4-Naphthoquinone	29.52 ± 1.0	19.29 ± 1.1	8.78 ± 1.1	0.30 ± 0.13	0.46 ± 0.14
**11a**	18.65 ± 1.1	9.16 ± 1.0	10.60 ± 1.2	0.57 ± 0.12	1.15 ± 0.15
**11b**	19.45 ± 1.1	15.05 ± 1.0	10.71 ± 1.3	0.55 ± 0.13	0.71 ± 0.14
**11c**	32.84 ± 1.1	16.40 ± 1.1	10.25 ± 1.1	0.31 ± 0.11	0.62 ± 0.12
**11d**	25.14 ± 1.2	20.12 ± 1.1	21.49 ± 1.2	0.85 ± 0.07	1.07 ± 0.08
**11e**	ND *	ND *	ND *	ND *	ND *
**11f**	65.70 ± 1.1	24.10 ± 1.1	6.92 ± 1.1	0.11 ± 0.16	0.29 ± 0.16
Doxorubicin	14.22 ± 1.1	2.76 ± 1.2	1.49 ± 1.3	0.10 ± 0.09	0.54 ± 0.10

* ND: not determined due to the low solubility of this compound at concentrations >25 µM.

**Table 2:** Physicochemical parameter values for compounds **11a–f** using Swiss ADME server.

Compound	MW ^a^	nHA ^b^	nAHA ^c^	nRotB ^d^	nHBA ^e^	nHBD ^f^	MR ^g^	TPSA ^h^	MlogP ^i^	ESOL ^j^
**11a**	401.41	30	16	5	5	0	113.44	82.44	1.75	MS
**11b**	449.45	34	22	5	5	0	128.71	82.44	2.63	MS
**11c**	443.49	33	16	8	5	0	127.86	82.44	2.37	MS
**11d**	373.36	28	16	3	5	1	104.32	93.44	1.33	MS
**11e**	435.43	33	22	4	5	1	124.0	93.44	2.16	MS
**11f**	415.44	31	16	6	5	1	118.74	93.44	1.96	MS

(**a**) Molecular weight (MW); (**b**) number of heavy atoms (nHA); (**c**) number of aromatic heavy atoms (nAHA); (**d**) number of rotatable bonds (nRotB); (**e**) number of hydrogen bond acceptors (nHBA); (**f**) number of hydrogen bond donors (nHBD); (**g**) molecular refractivity (MR); (**h**) topological polar surface area (TPSA); (**i**) octanol/water partition coefficient (MlogP, logP by the method of Moriguchi); (**j**) estimated water solubility (ESOL) with MS moderately soluble and S representing soluble.

## Data Availability

The raw data necessary to support the results of this article will be promptly made available by the authors upon request.

## Supplementary Materials

The following supporting information can be downloaded at: https://www.mdpi.com/article/10.3390/ijms25126490/s1.
