# Supplementary material for: Naphthoquinone-Quinolone Hybrids with Antitumor Effects on Breast Cancer Cell Lines—From the Synthesis to 3D-Cell Culture Effects
Source: Int J Mol Sci. 2024 Jun 12;25(12):6490. doi: 10.3390/ijms25126490 (PMC11203957; doi:10.3390/ijms25126490)
Supplement: Supplementary file 1 [file ijms-25-06490-s001.zip › ijms-2988715-supplementary.pdf]

**Supplementary Material**

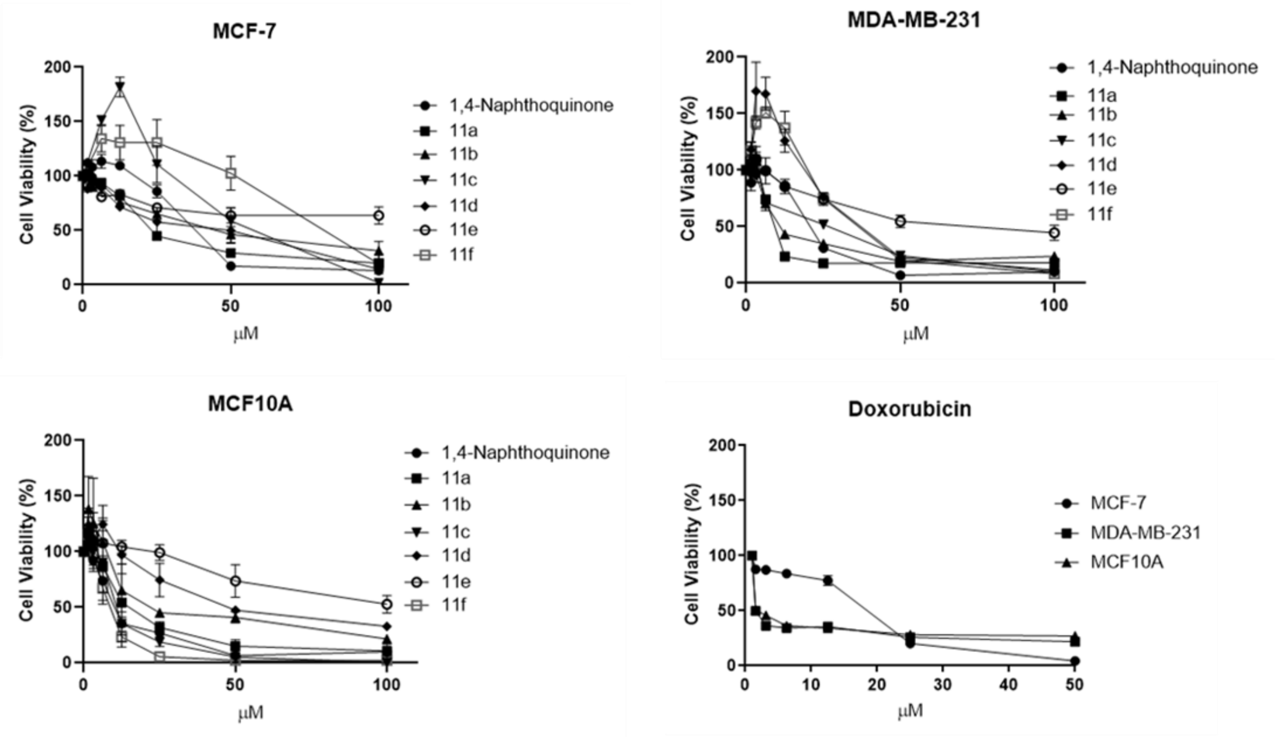


**Figure S1.** Dose-response curves for compounds 11a-f, 1,4-naphthoquinone, and doxorubicin on breast cancer cells (MCF-7 and MDA-MB-231) and breast cell line (MCF10A).

**Ethyl 6-(1,4-dioxo-1,4-dihydronaphthalen-2-yl)-1-ethyl-4-oxo-1,4-dihydroquinoline-3-carboxylate (11a)**

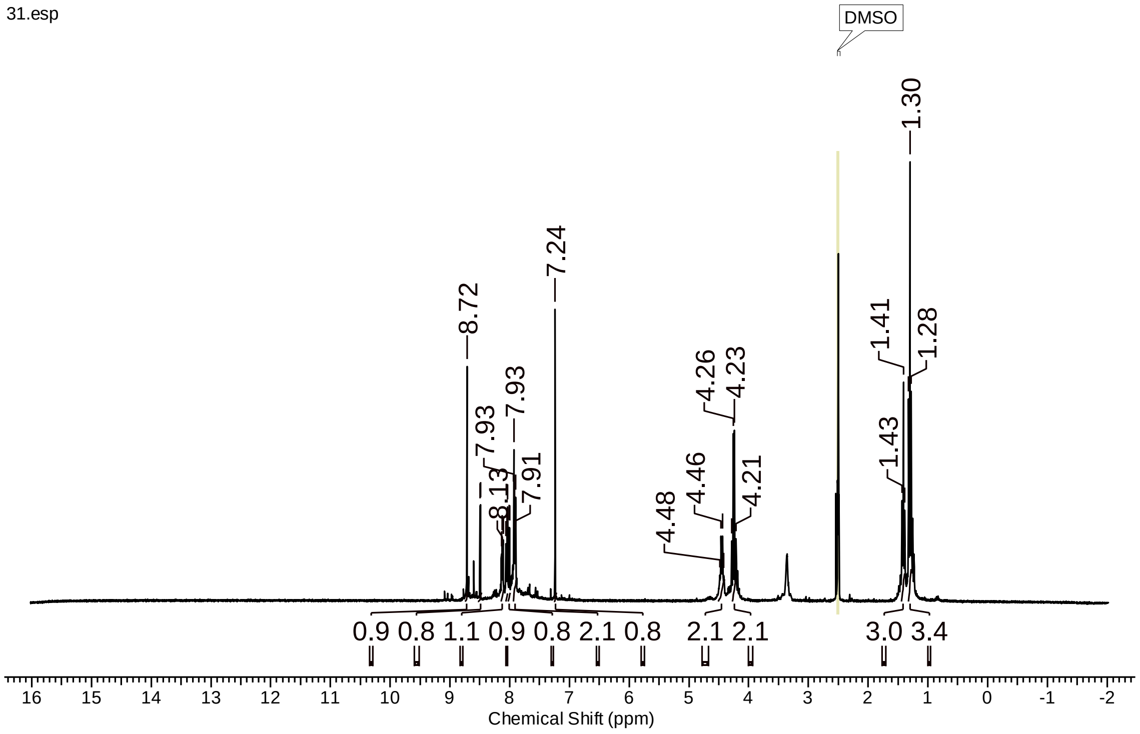


**Figure S2.** $^{1}H$ NMR spectrum of derivative **11a** (DMSO-$d_6$, 300.00 MHz).

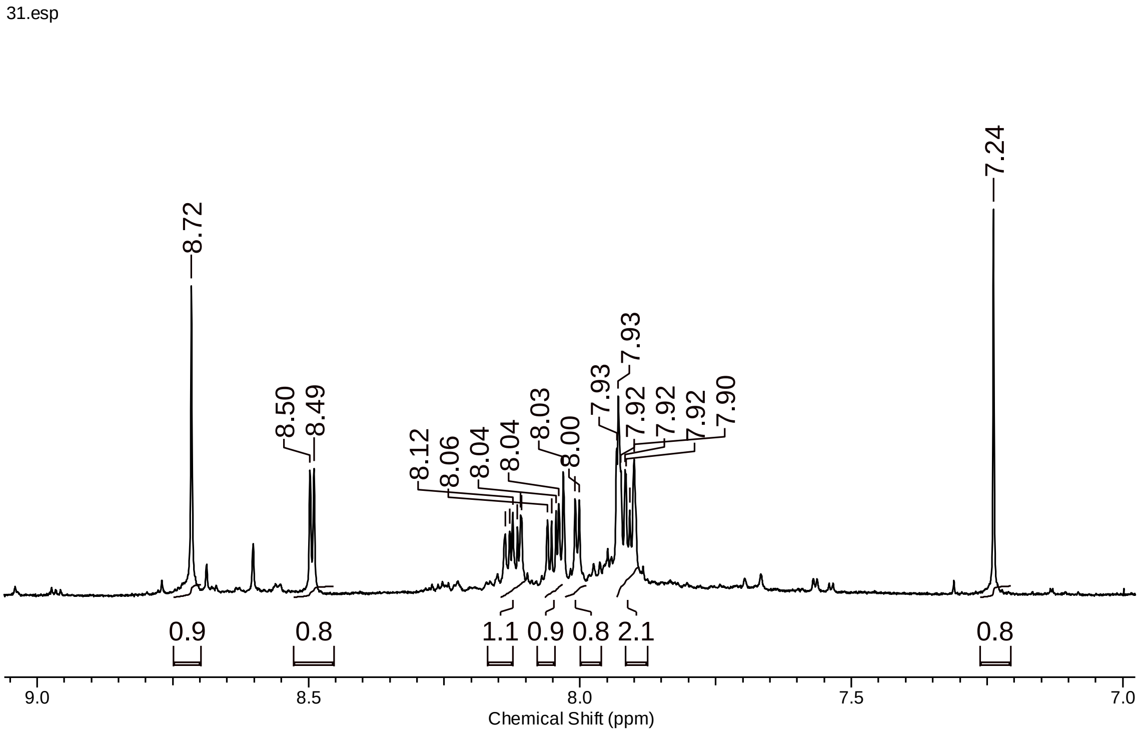


**Figure S3.** Expansion of the $^{1}H$ NMR spectrum of derivative **11a** (DMSO-$d_6$, 300.00 MHz).

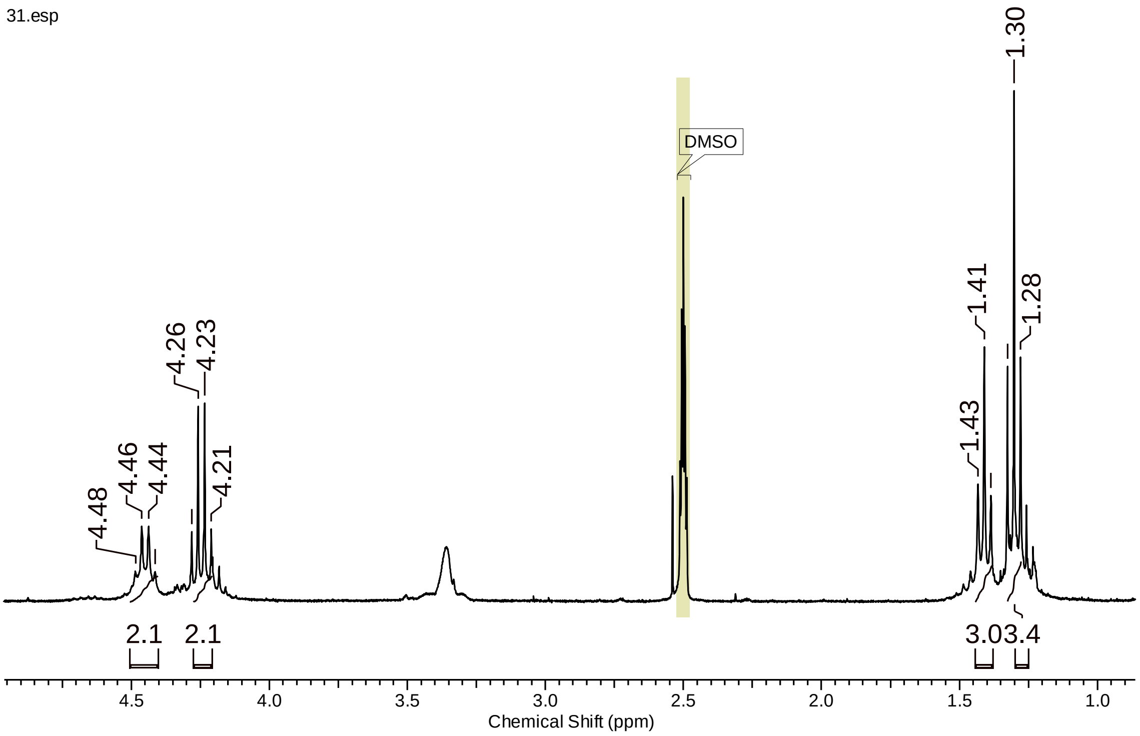


**Figure S4.** Expansion of the $^{1}$H NMR spectrum of derivative **11a** (DMSO-$d_6$, 300.00 MHz).

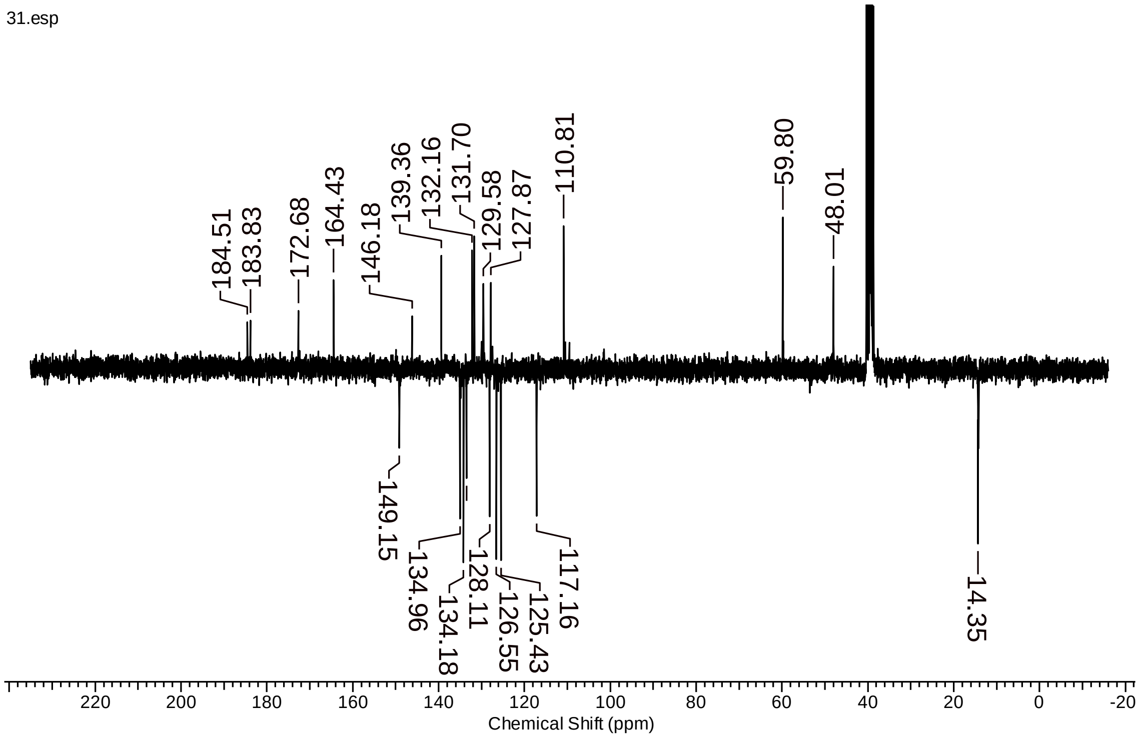


**Figure S5.** $^{13}$C-APT NMR spectrum of derivative **11a** (DMSO-$d_6$, 75.0 MHz).

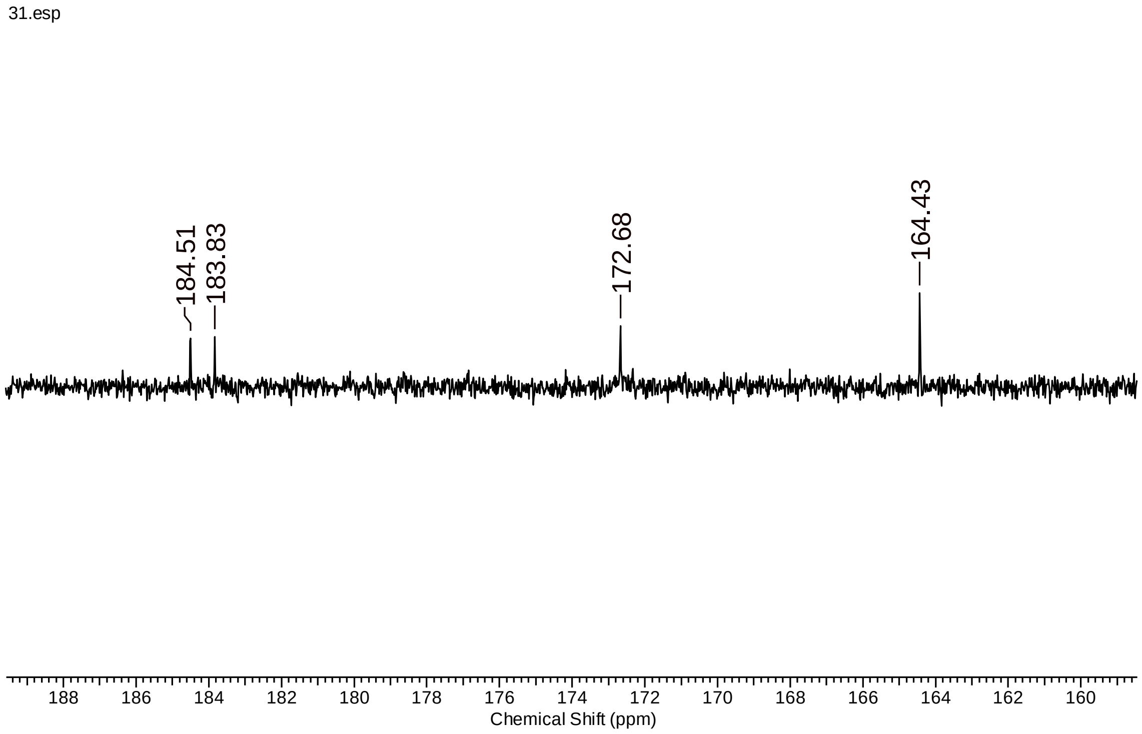


**Figure S6.** Expansion of the $^{13}$C-APT NMR spectrum of derivative **11a** (DMSO-$d_6$, 75.0 MHz).

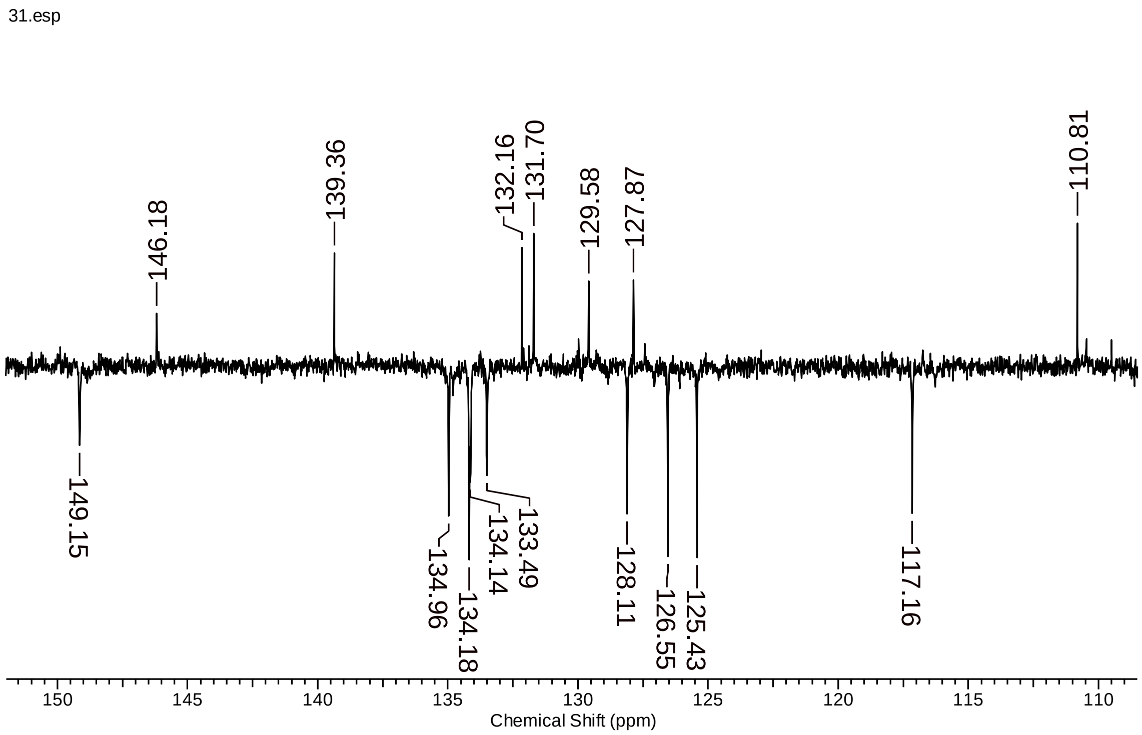


**Figure S7.** Expansion of the $^{13}$C-APT NMR spectrum of derivative **11a** (DMSO-$d_6$, 75.0 MHz).

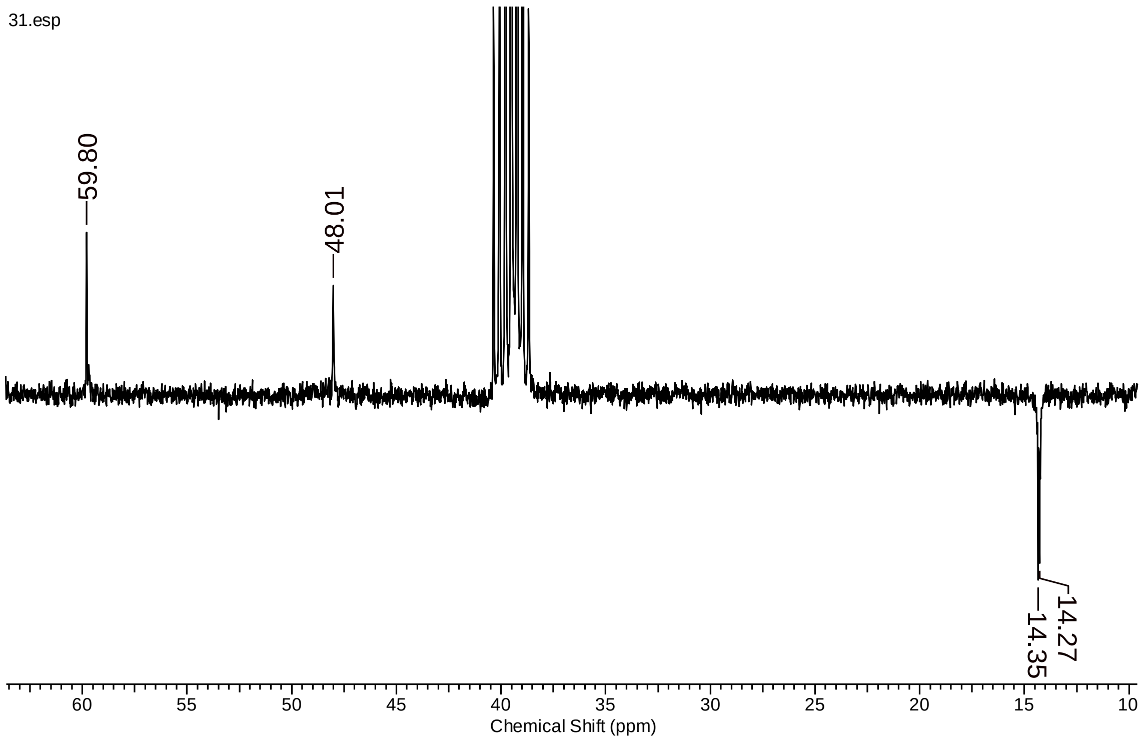


**Figure S8.** Expansion of the $^{13}$C-APT NMR spectrum of derivative **11a** (DMSO-$d_6$, 75.0 MHz).

## Ethyl 1-benzyl-6-(1,4-dioxo-1,4-dihydronaphthalen-2-yl)-4-oxo-1,4-dihydroquinoline-3-carboxylate (11b)

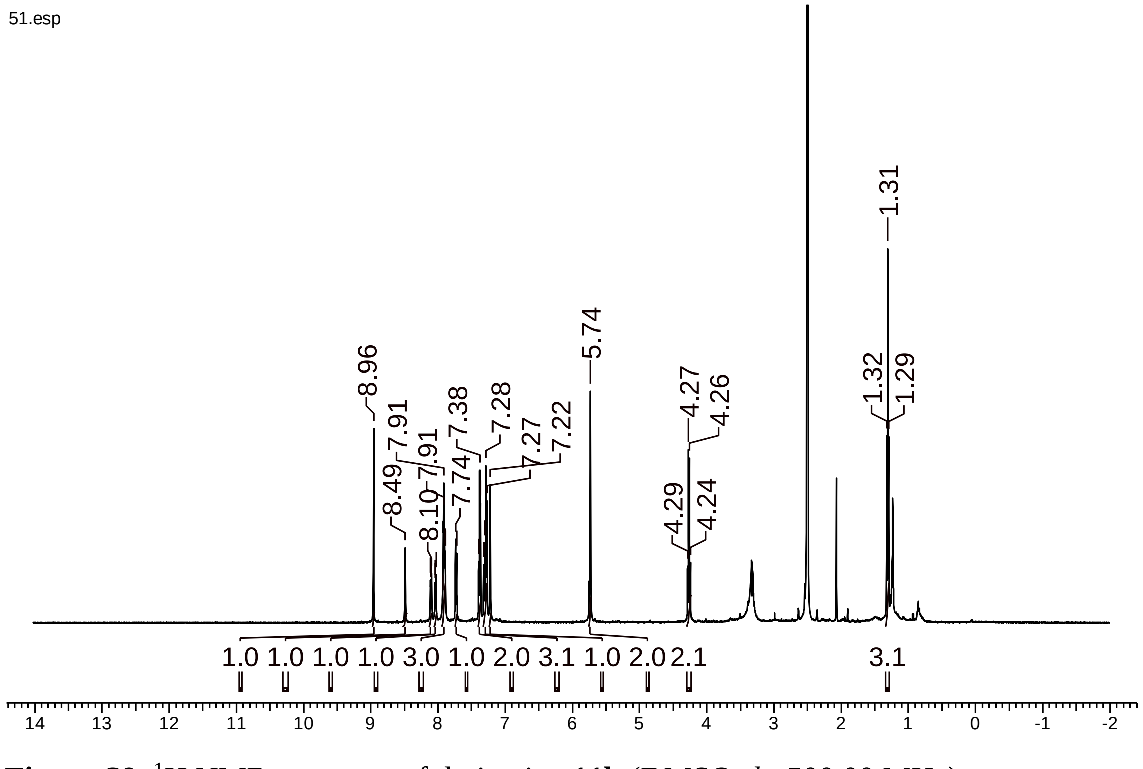


**Figure S9.** $^1$H NMR spectrum of derivative **11b** (DMSO-$d_6$, 500.00 MHz).

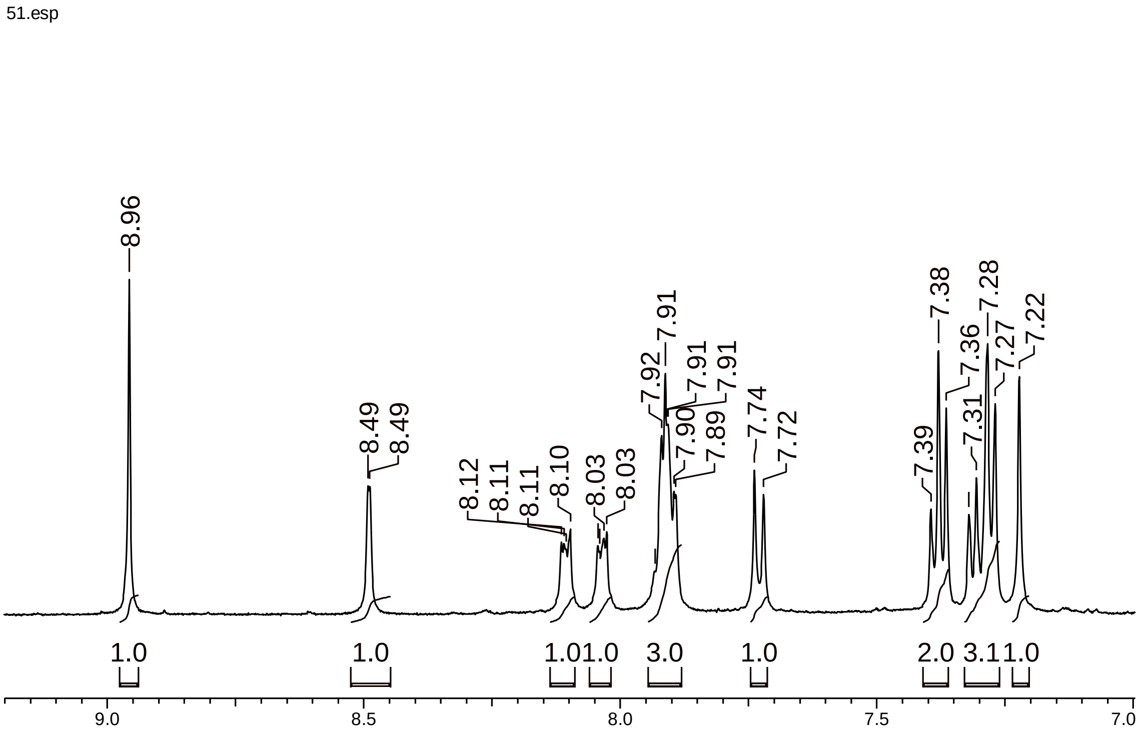


**Figure S10.** Expansion of the $^{1}H$ NMR spectrum of derivative **11b** (DMSO-$d_6$, 500.00 MHz).

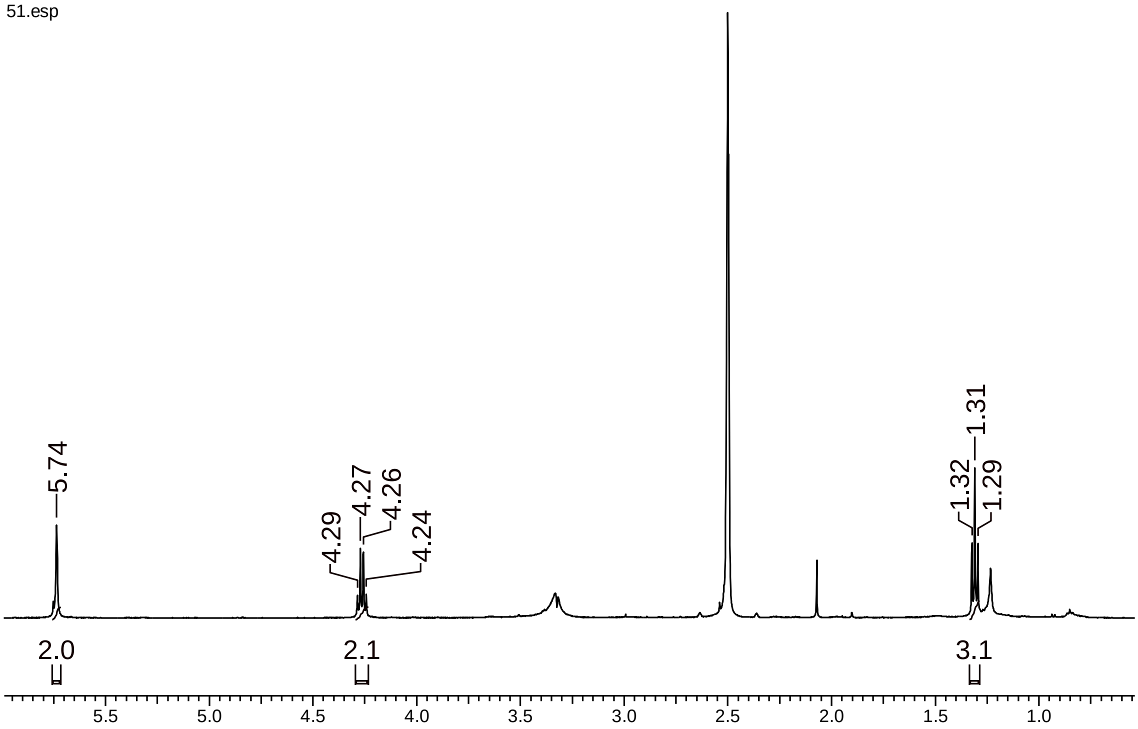


**Figure S11.** Expansion of the $^{1}H$ NMR spectrum of derivative **11b** (DMSO-$d_6$, 500.00 MHz).

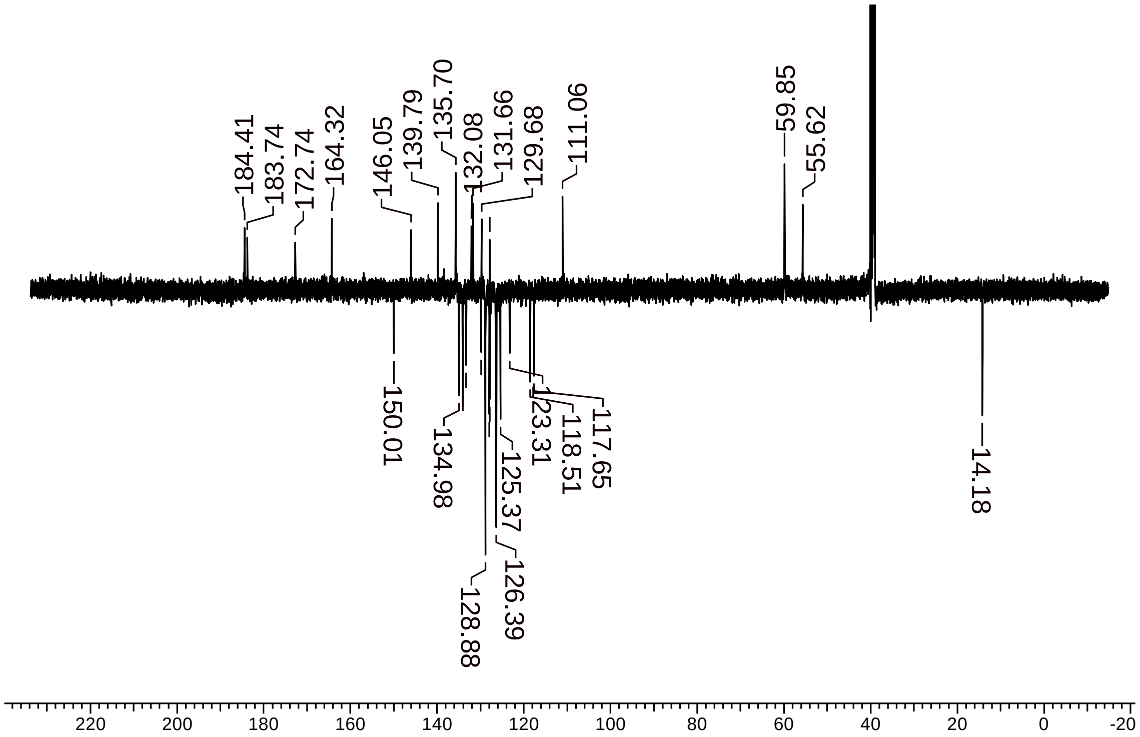


**Figure S12.** $^{13}$C-APT NMR spectrum of derivative **11b** (DMSO-$d_6$, 125.00 MHz).

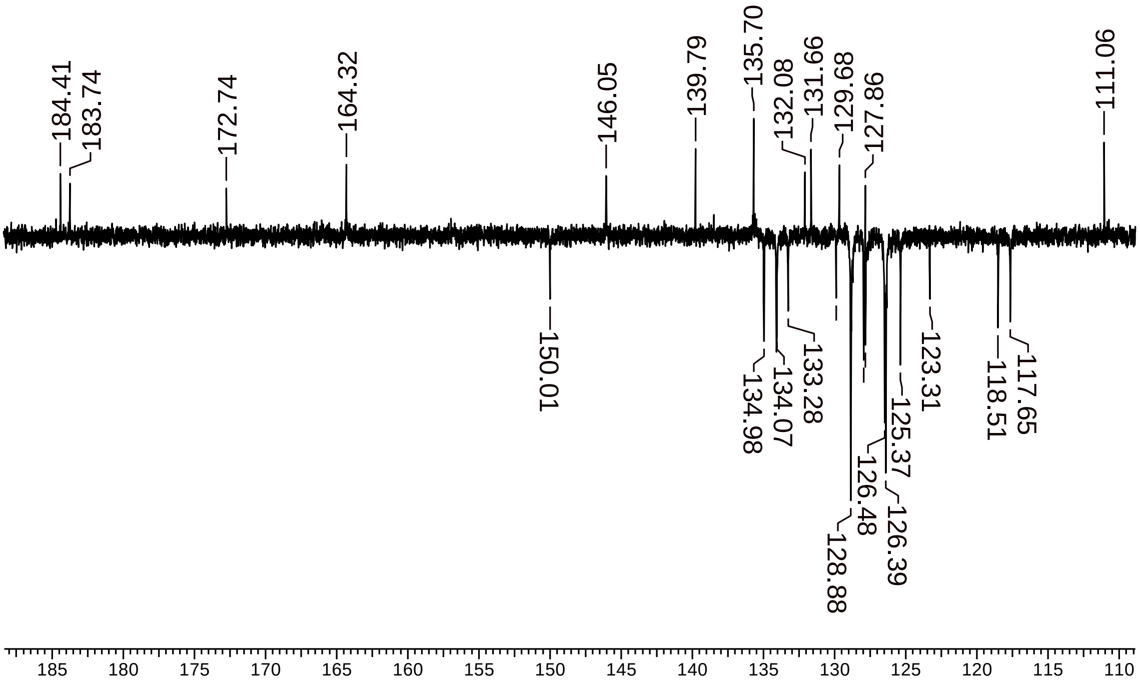


**Figure S13.** Expansion of the $^{13}$C-APT NMR spectrum of derivative **11b** (DMSO-$d_6$, 125.00 MHz).

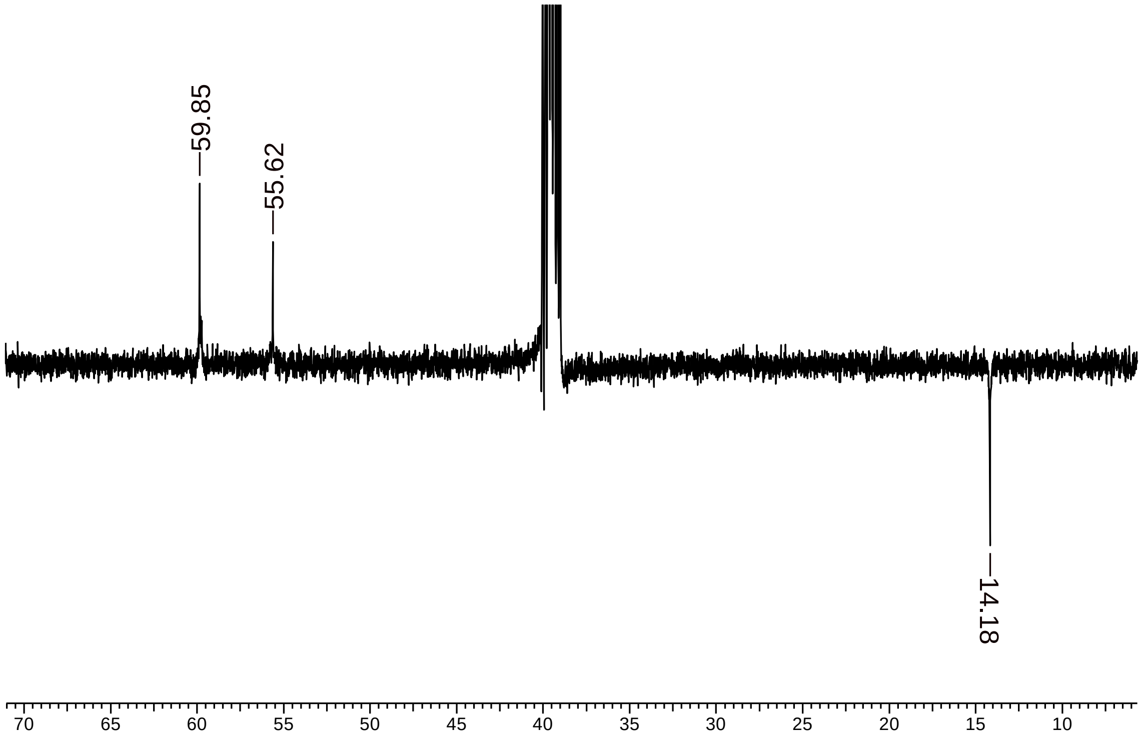


**Figure S14.** Expansion of the $^{13}$C-APT NMR spectrum of derivative **11b** (DMSO-$d_6$, 125.00 MHz).

**Ethyl 6-(1,4-dioxo-1,4-dihydronaphthalen-2-yl)-4-oxo-1-pentyl-1,4-dihydroquinoline-3-carboxylate (11c)**

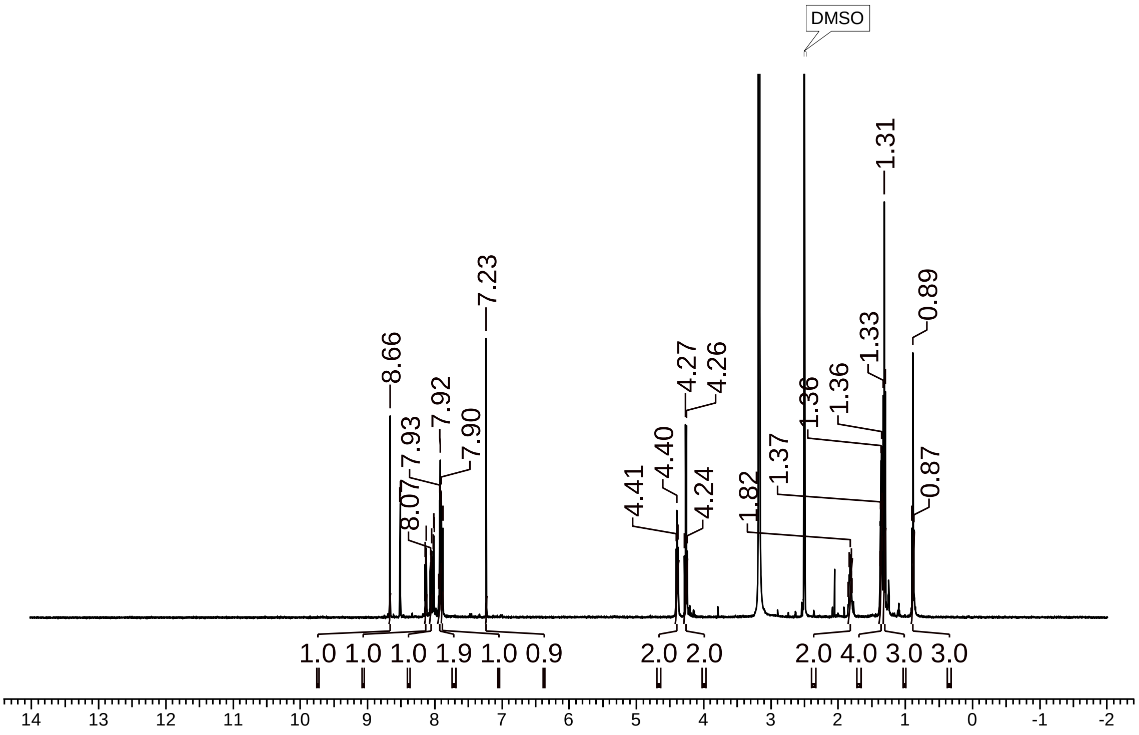


**Figure S15.** $^{1}$H NMR spectrum of derivative **11c** (DMSO-$d_6$, 500.00 MHz).

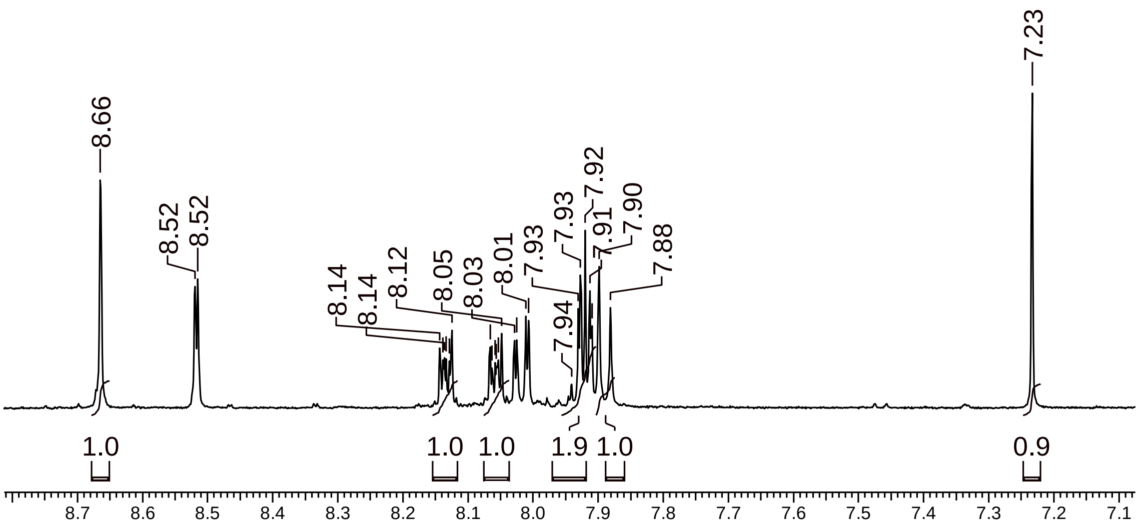


**Figure S16.** Expansion of the $^{1}$H NMR spectrum of derivative **11c** (DMSO-$d_6$, 500.00 MHz).

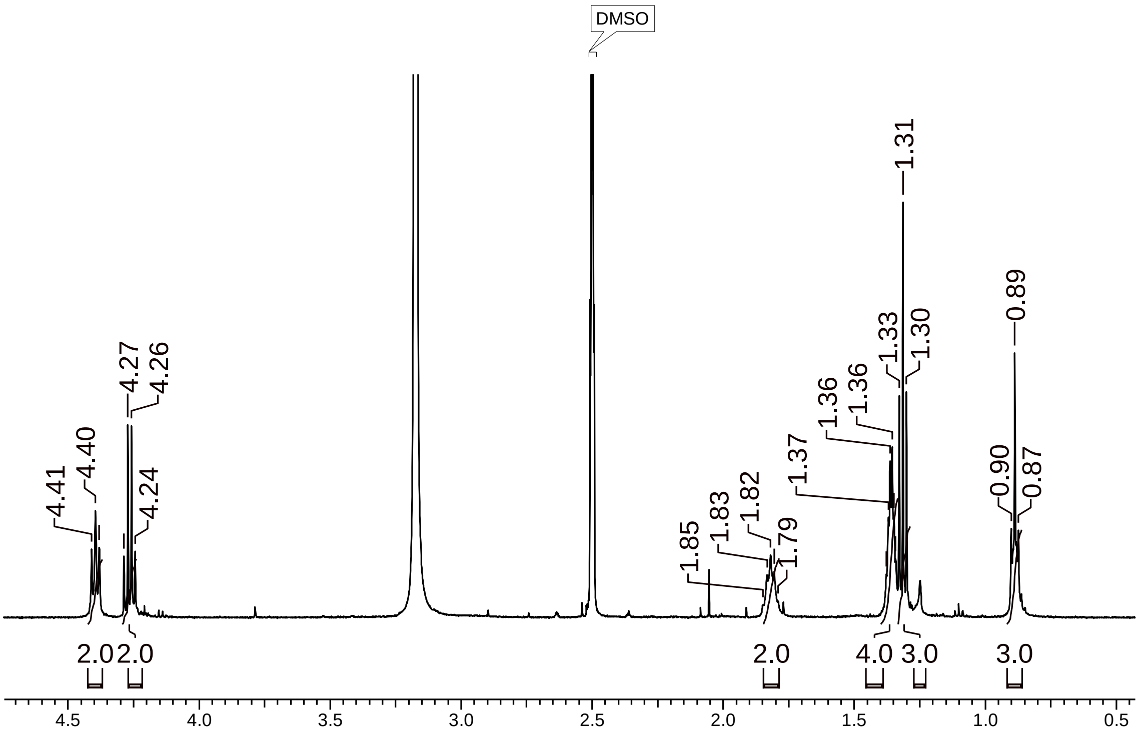


**Figure S17.** Expansion of the $^{1}$H NMR spectrum of derivative **11c** (DMSO-$d_6$, 500.00 MHz).

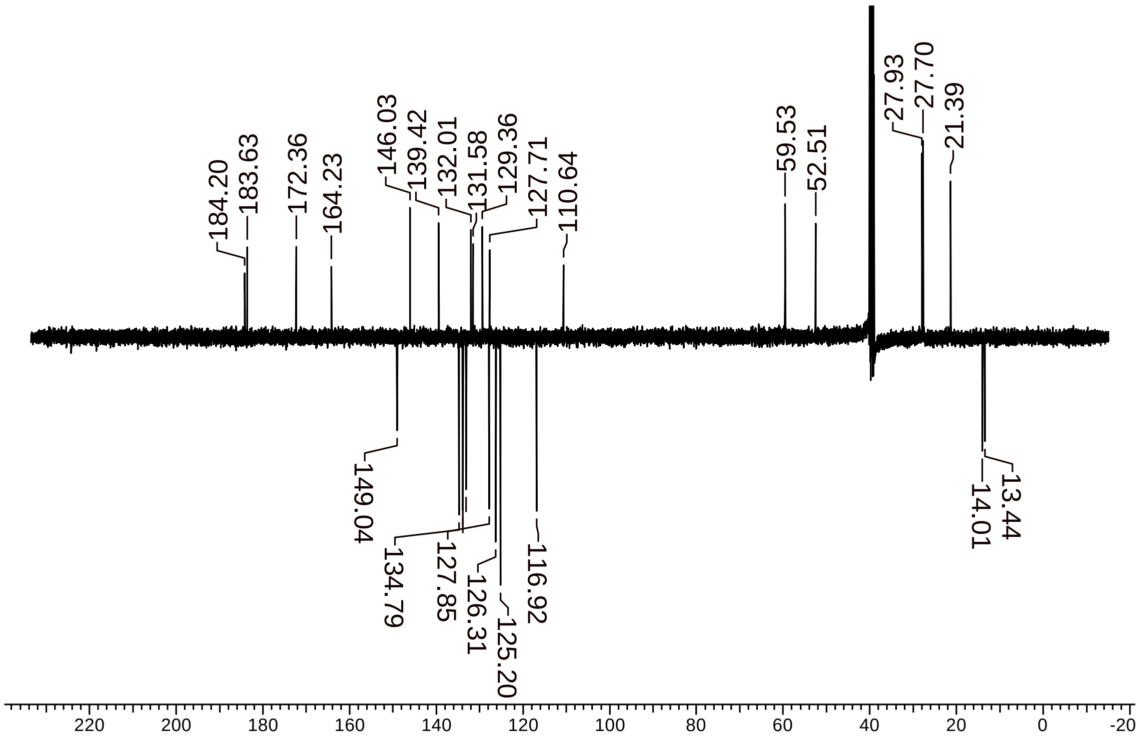


**Figure S18.** $^{13}$C-APT NMR spectrum of derivative **11c** (DMSO-$d_6$, 125.00 MHz).

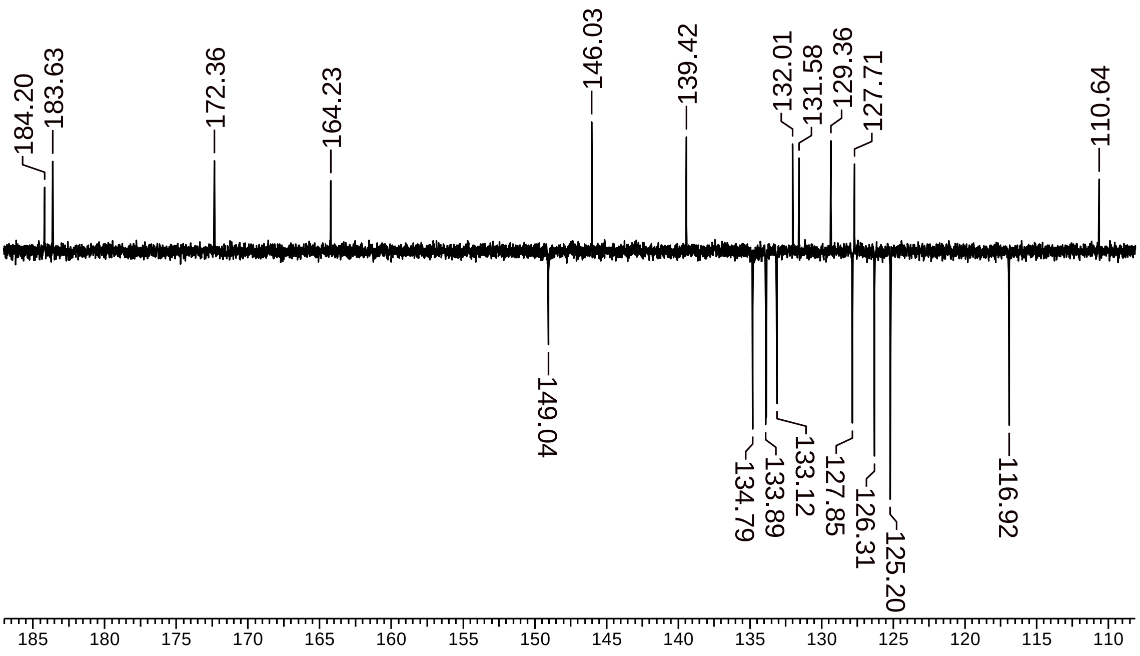


**Figure S19.** Expansion of the $^{13}$C-APT NMR spectrum of derivative **11c** (DMSO-$d_6$, 125.00 MHz).

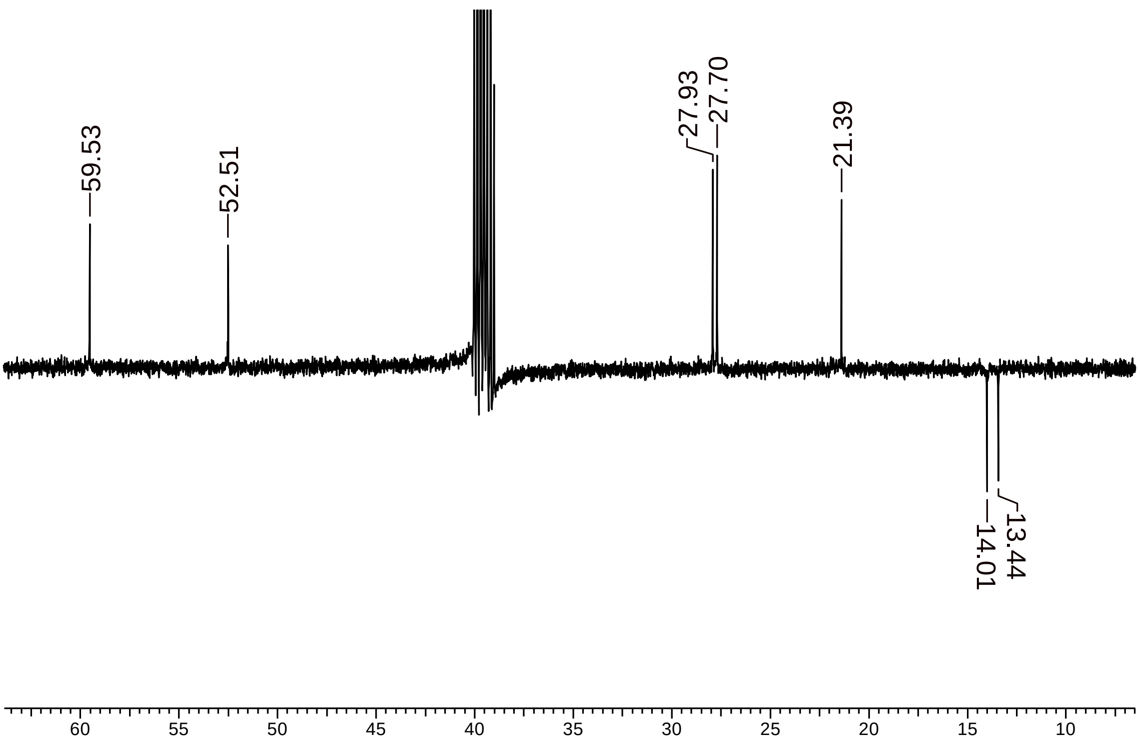


**Figure S20.** Expansion of the $^{13}$C-APT NMR spectrum of derivative **11c** (DMSO-$d_6$, 125.00 MHz).

**6-(1,4-dioxo-1,4-dihydronaphthalen-2-yl)-1-ethyl-4-oxo-1,4-dihydroquinoline-3-carboxylic acid (11d)**

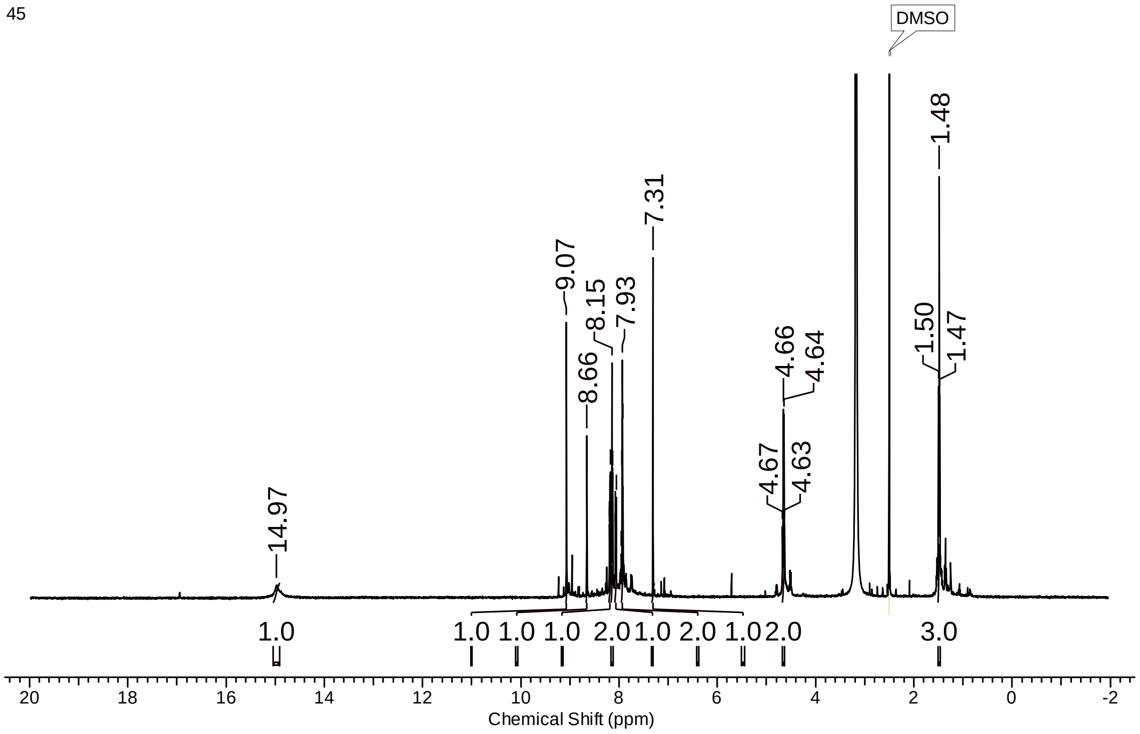


**Figure S21.** $^1$H NMR spectrum of derivative **11d** (DMSO-$d_6$, 500.00 MHz).

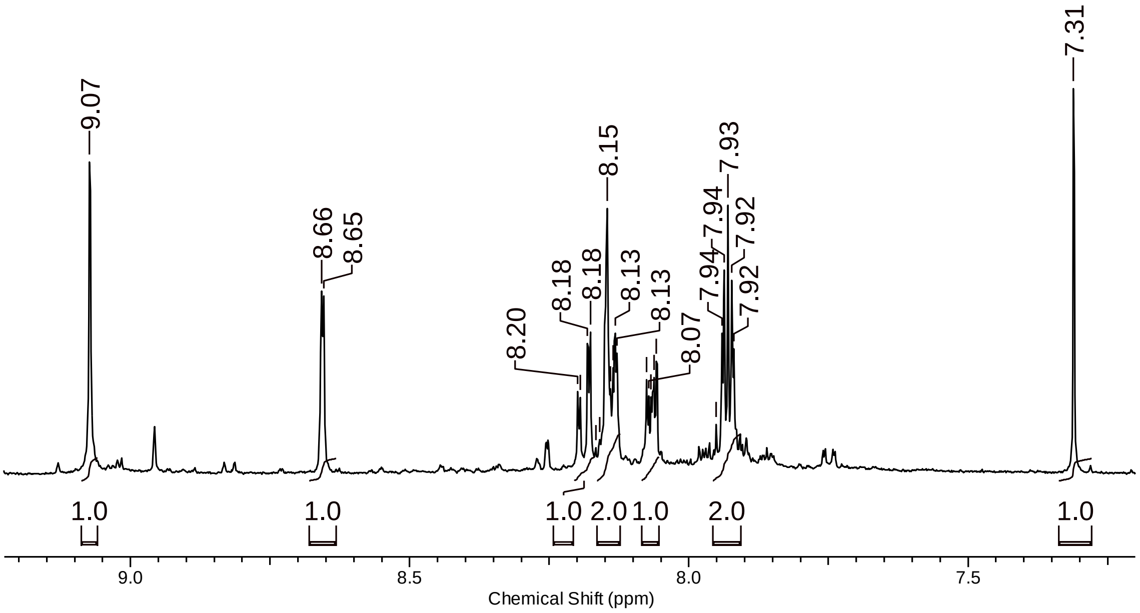


**Figure S22.** Expansion of the $^{1}H$ NMR spectrum of derivative **11d** (DMSO-$d_6$, 500.00 MHz).

45

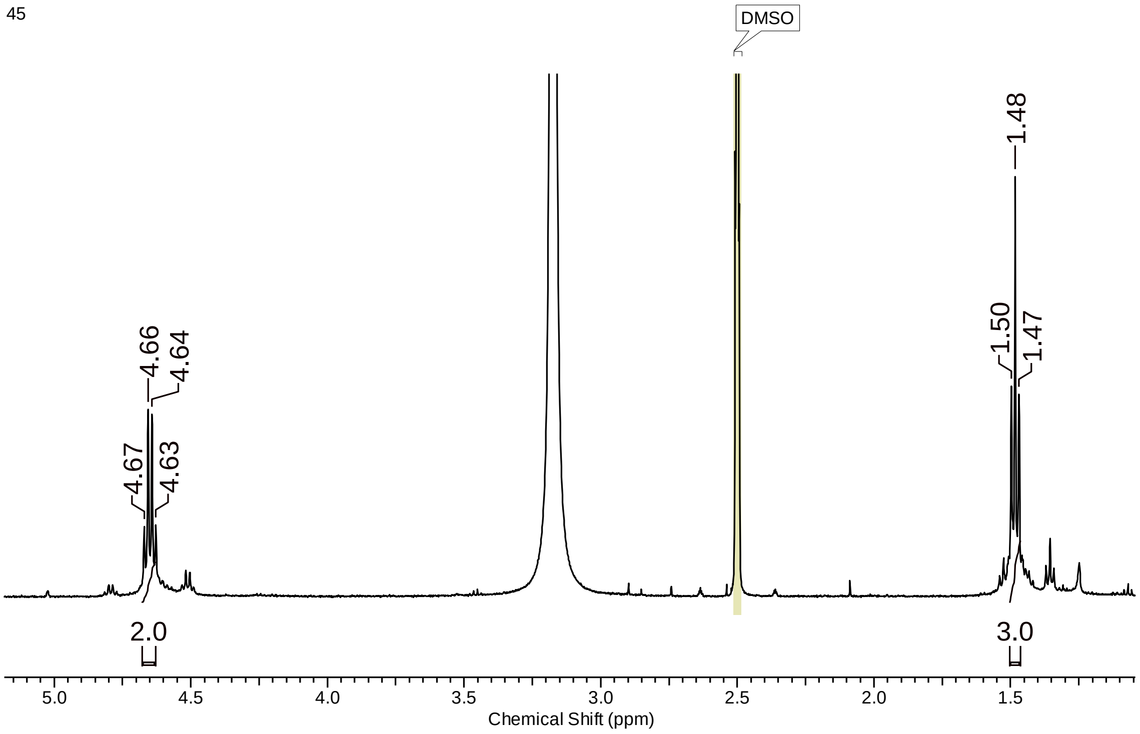


**Figure S23.** Expansion of the $^{1}H$ NMR spectrum of derivative **11d** (DMSO-$d_6$, 500.00 MHz).

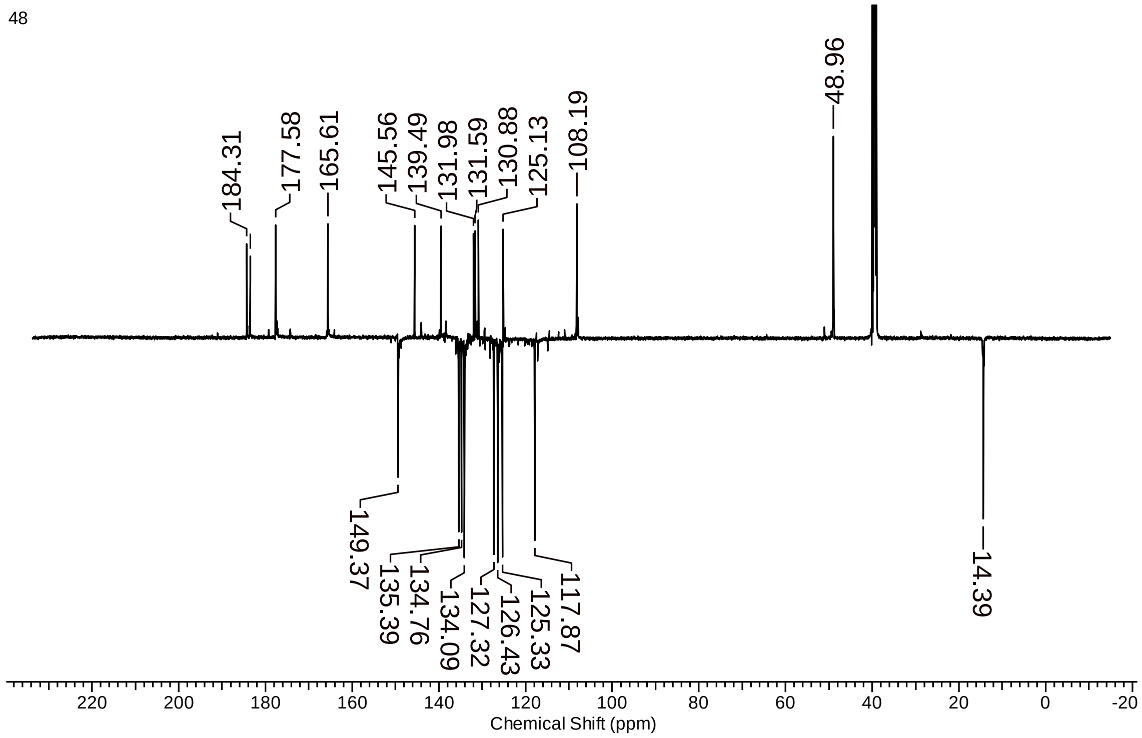


**Figure S24.** $^{13}$C-APT NMR spectrum of derivative **11d** (DMSO-$d_6$, 125.00 MHz).

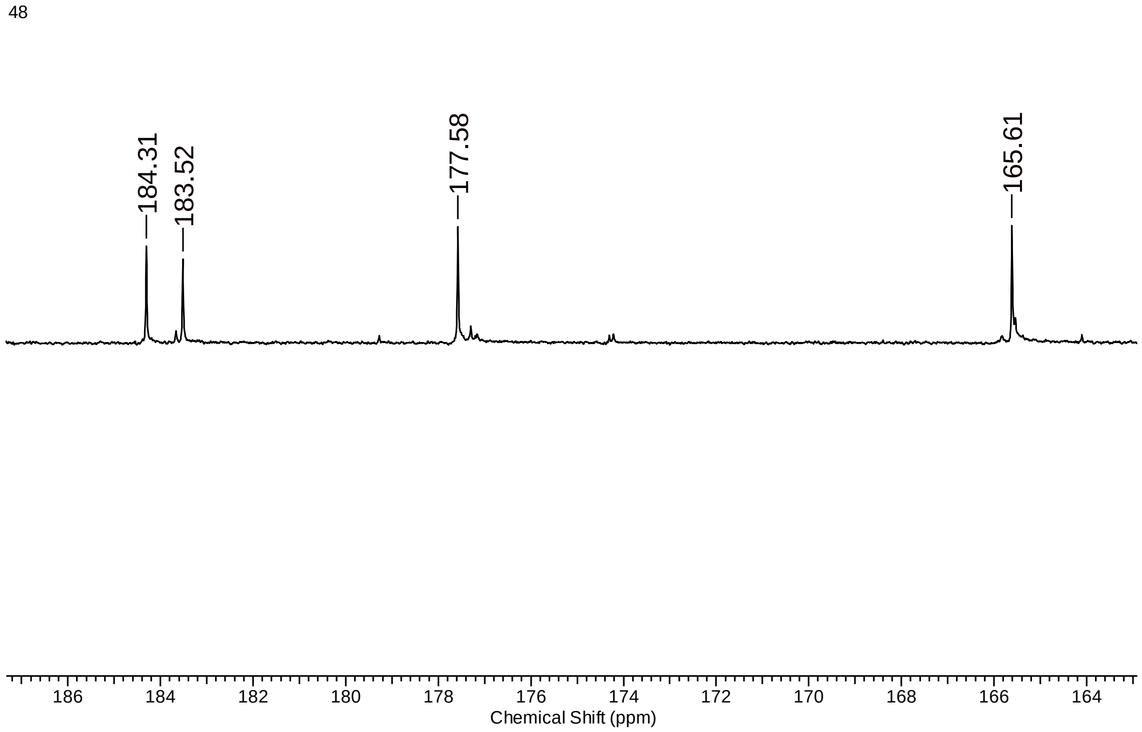


**Figure S25.** Expansion of the $^{13}$C-APT NMR spectrum of derivative **11d** (DMSO-$d_6$, 125.00 MHz).

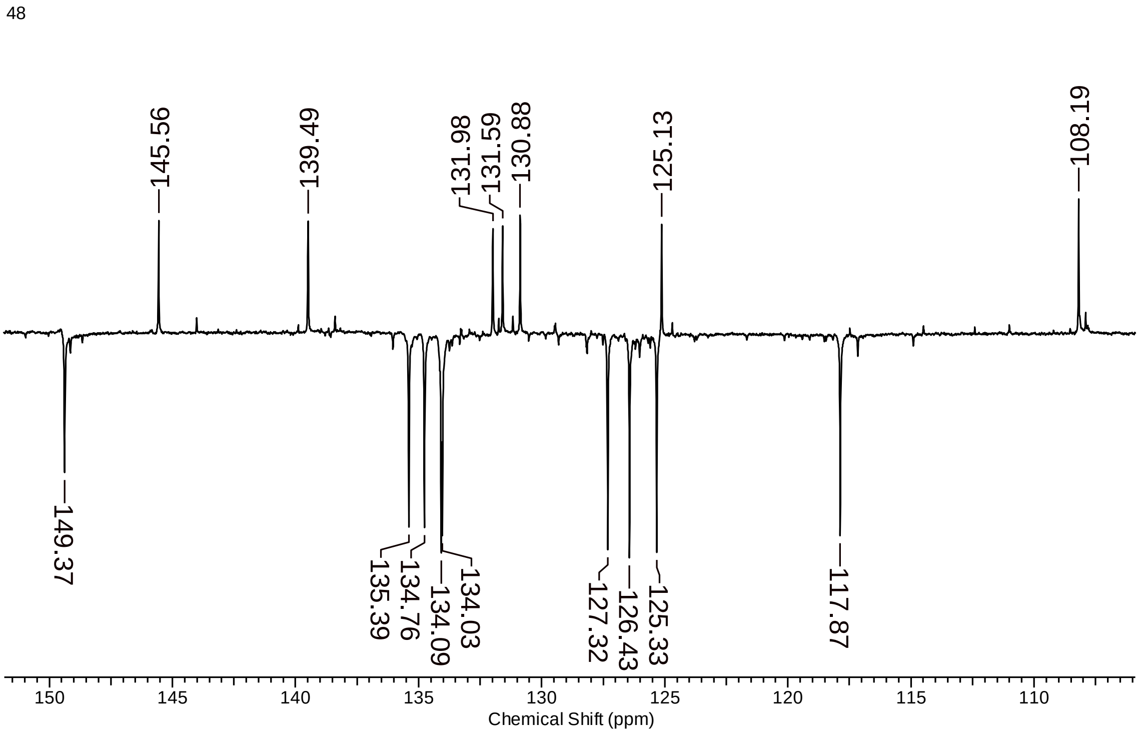


**Figure S26.** Expansion of the $^{13}C$-APT NMR spectrum of derivative **11d** (DMSO-$d_6$, 125.00 MHz).

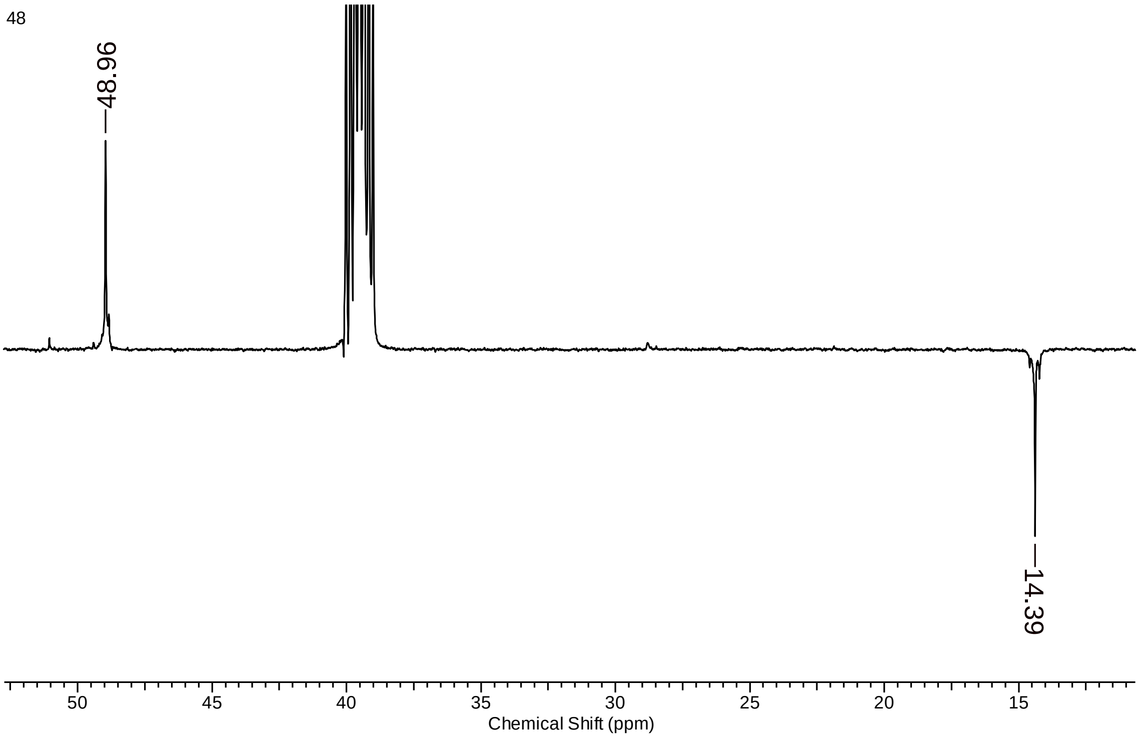


**Figure S27.** Expansion of the $^{13}C$-APT NMR spectrum of derivative **11d** (DMSO-$d_6$, 125.00 MHz).

**1-benzyl-6-(1,4-dioxo-1,4-dihydronaphthalen-2-yl)-4-oxo-1,4-dihydroquinoline-3-carboxylic acid (11e)**

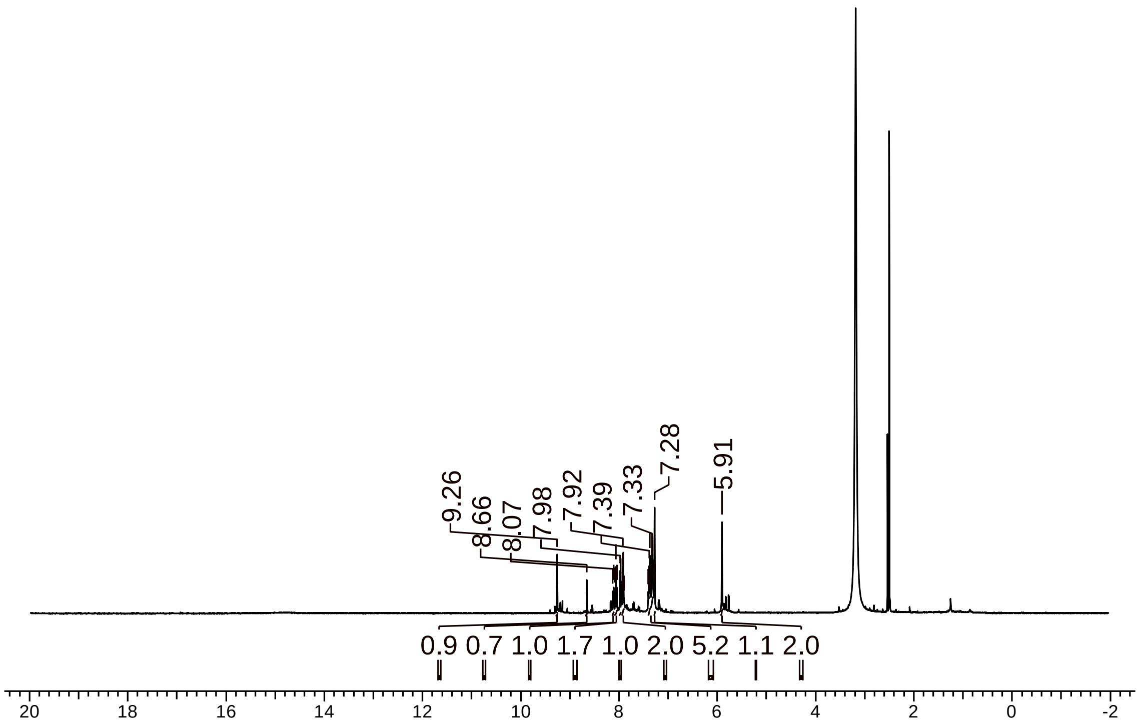

**Figure S28.** $^{1}$H NMR spectrum of derivative **11e** (DMSO-$d_6$, 500.00 MHz).

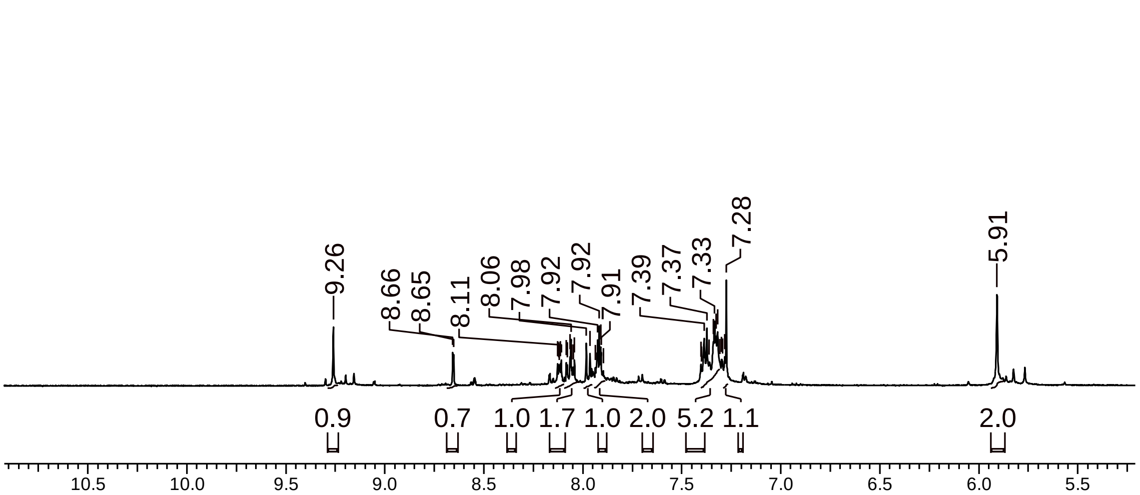

**Figure S29.** Expansion of the $^{1}$H NMR spectrum of derivative **11e** (DMSO-$d_6$, 500.00 MHz).

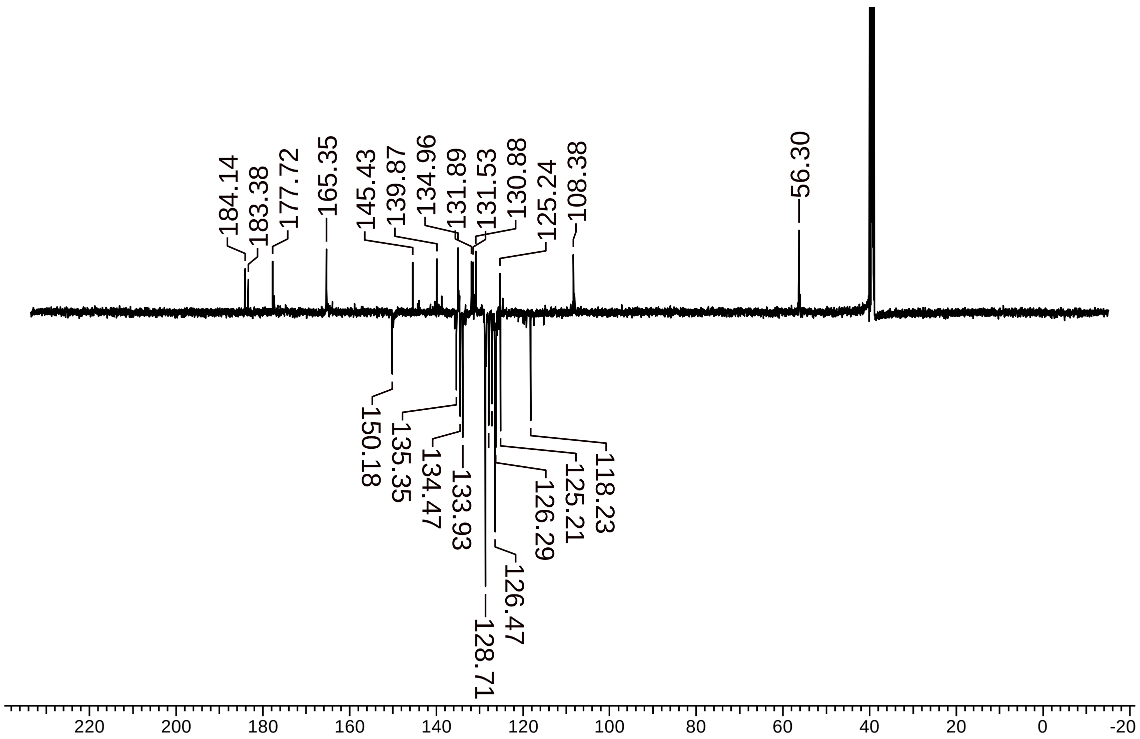


**Figure S30.** Expansion of the $^{13}$C-APT NMR spectrum of derivative **11**e (DMSO-$d_6$, 125.00 MHz).

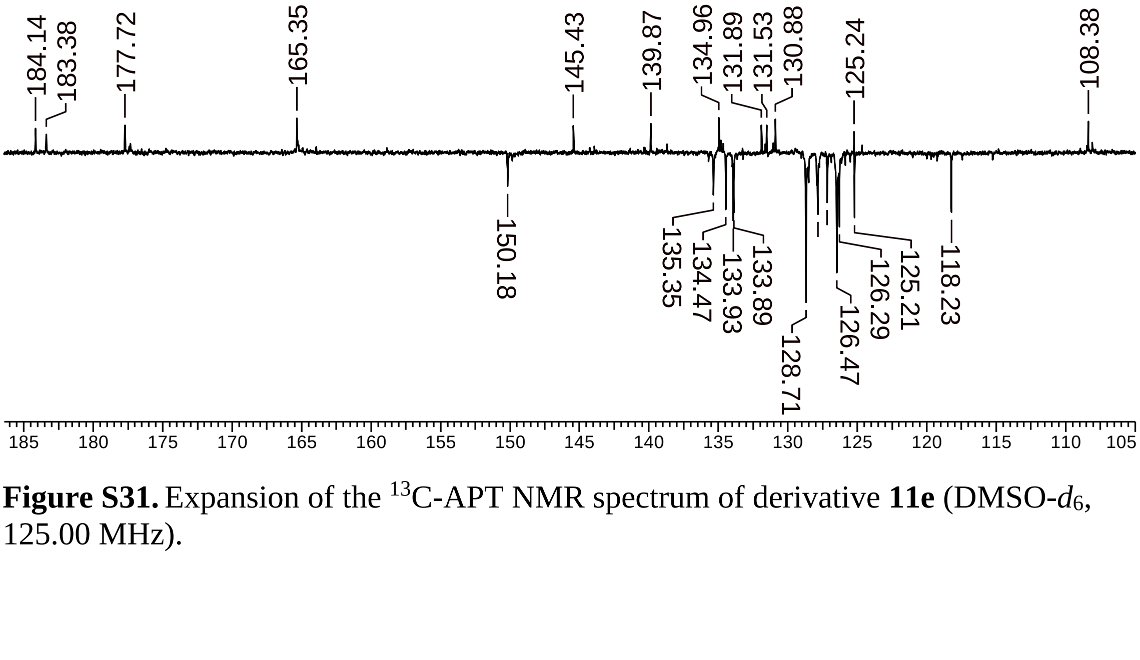


**Figure S31.** Expansion of the $^{13}$C-APT NMR spectrum of derivative **11e** (DMSO-$d_6$, 125.00 MHz).

**6-(1,4-dioxo-1,4-dihydronaphthalen-2-yl)-4-oxo-1-pentyl-1,4-dihydroquinoline-3-carboxylic acid (11f)**

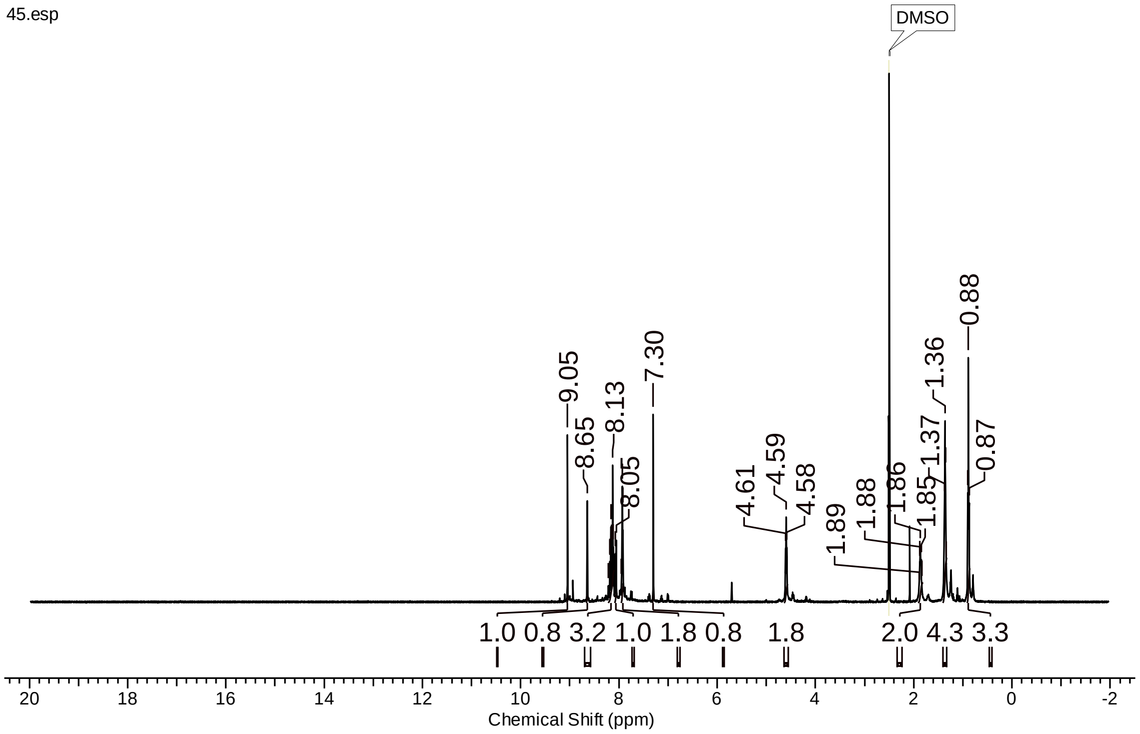


**Figure S32.** $^1$H NMR spectrum of derivative **11f** (DMSO-$d_6$, 500.00 MHz).

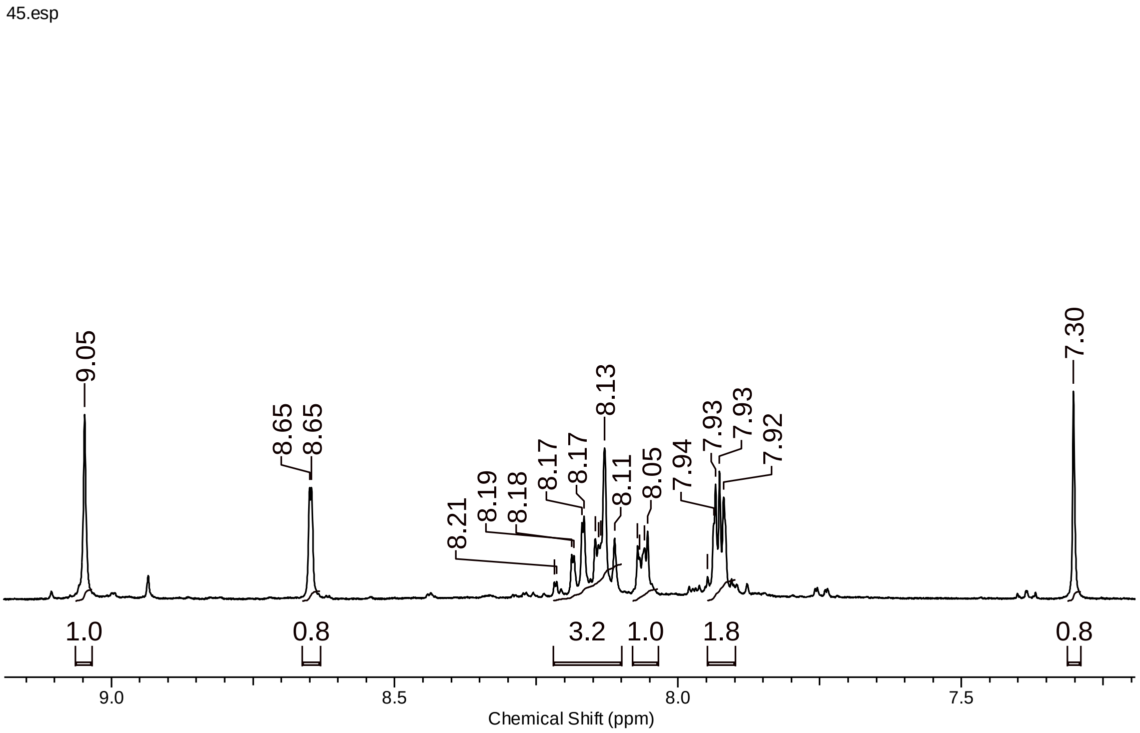


**Figure S33.** Expansion of the $^1$H NMR spectrum of derivative **11f** (DMSO-$d_6$, 500.00 MHz).

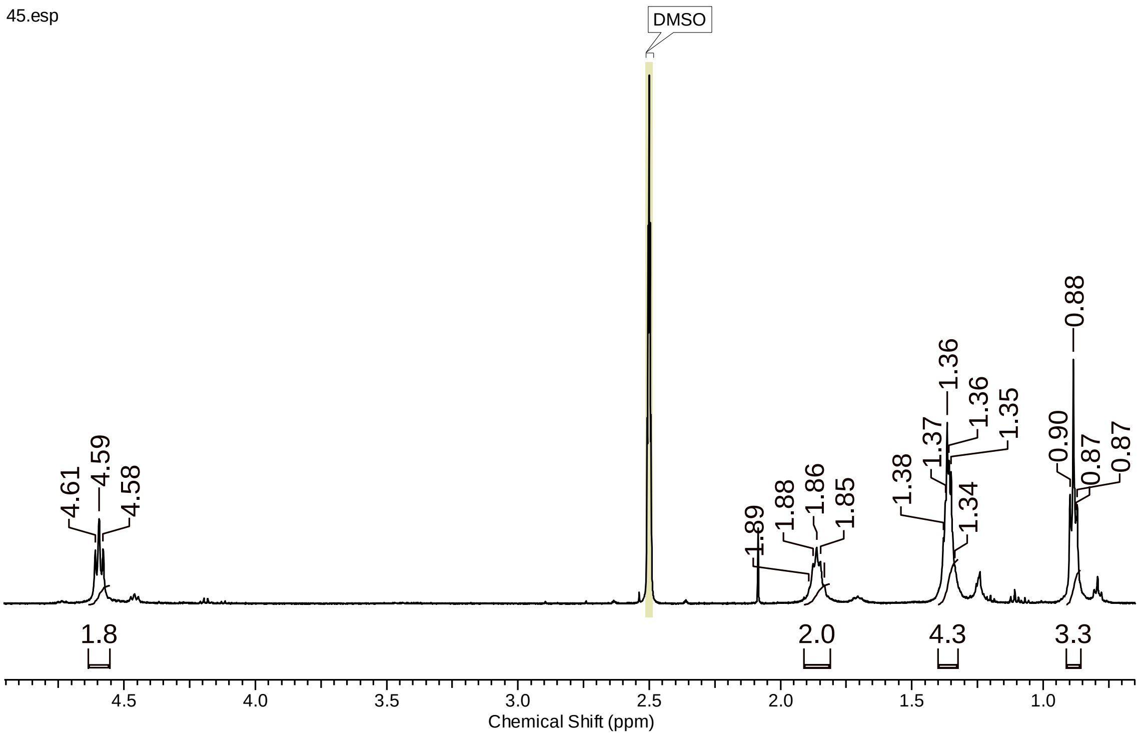


**Figure S34.** Expansion of the $^{1}H$ NMR spectrum of derivative **11f** (DMSO-$d_6$, 500.00 MHz).

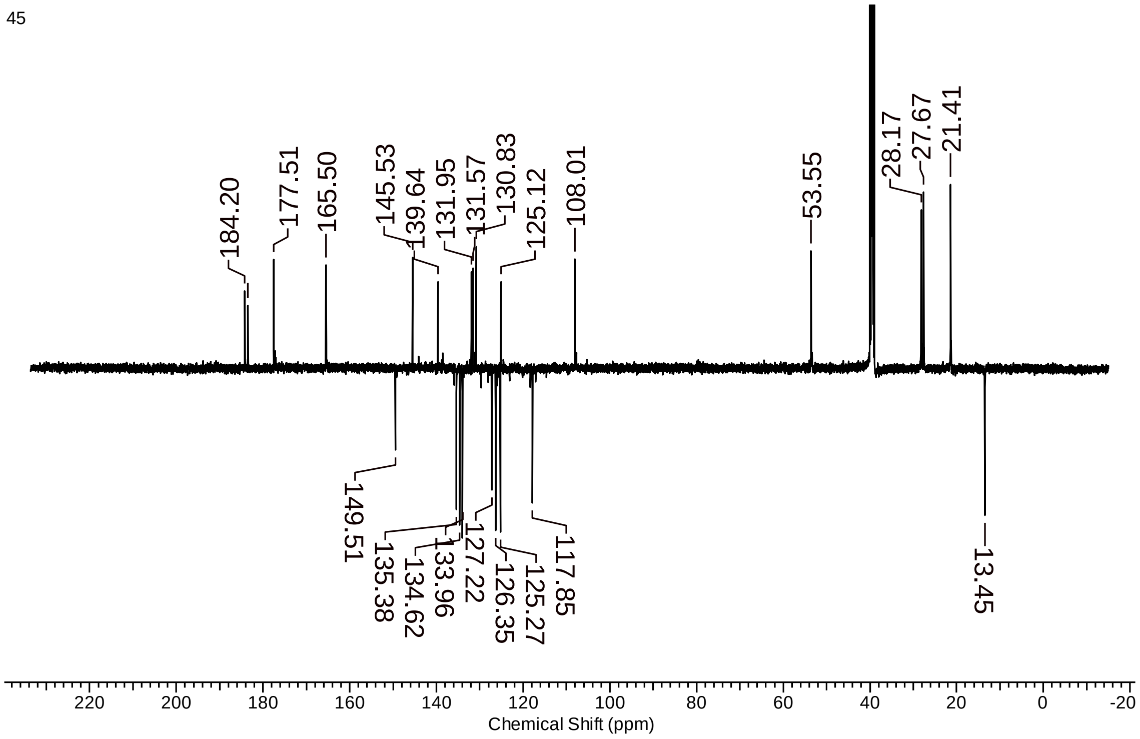


**Figure S35.** $^{13}C$-APT NMR spectrum of derivative **11f** (DMSO-$d_6$, 125.00 MHz).

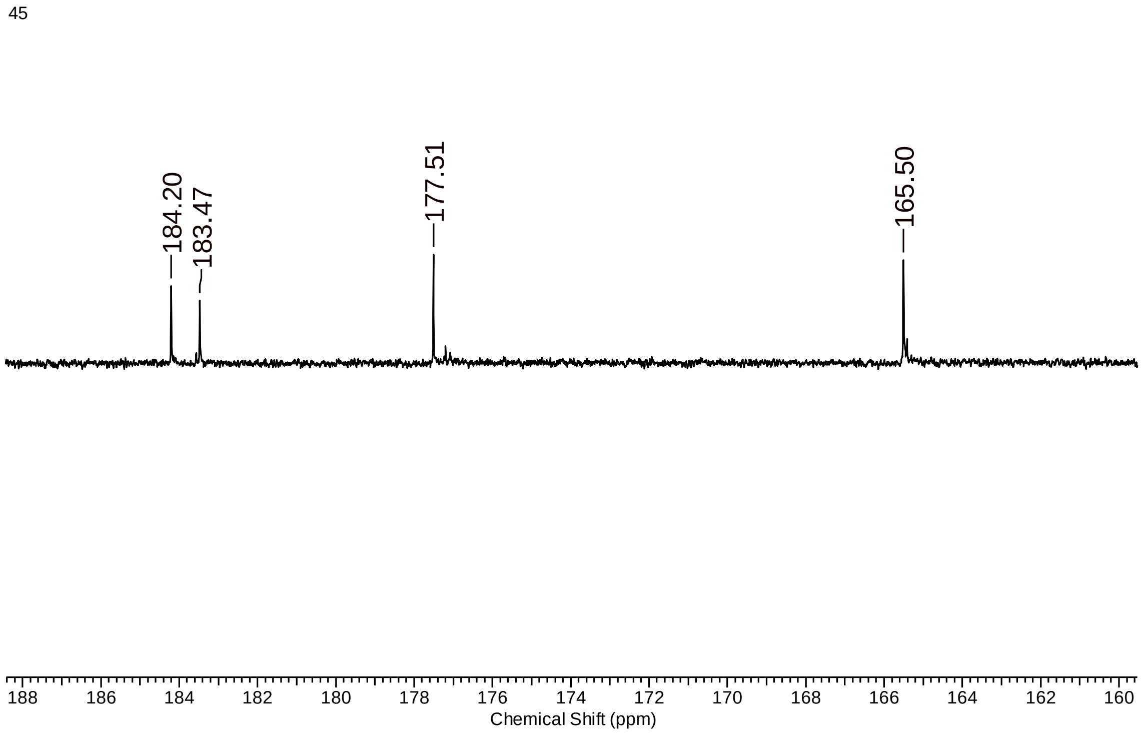


**Figure S36.** Expansion of the $^{13}C$-APT NMR spectrum of derivative **11f** (DMSO-$d_6$, 125.00 MHz).

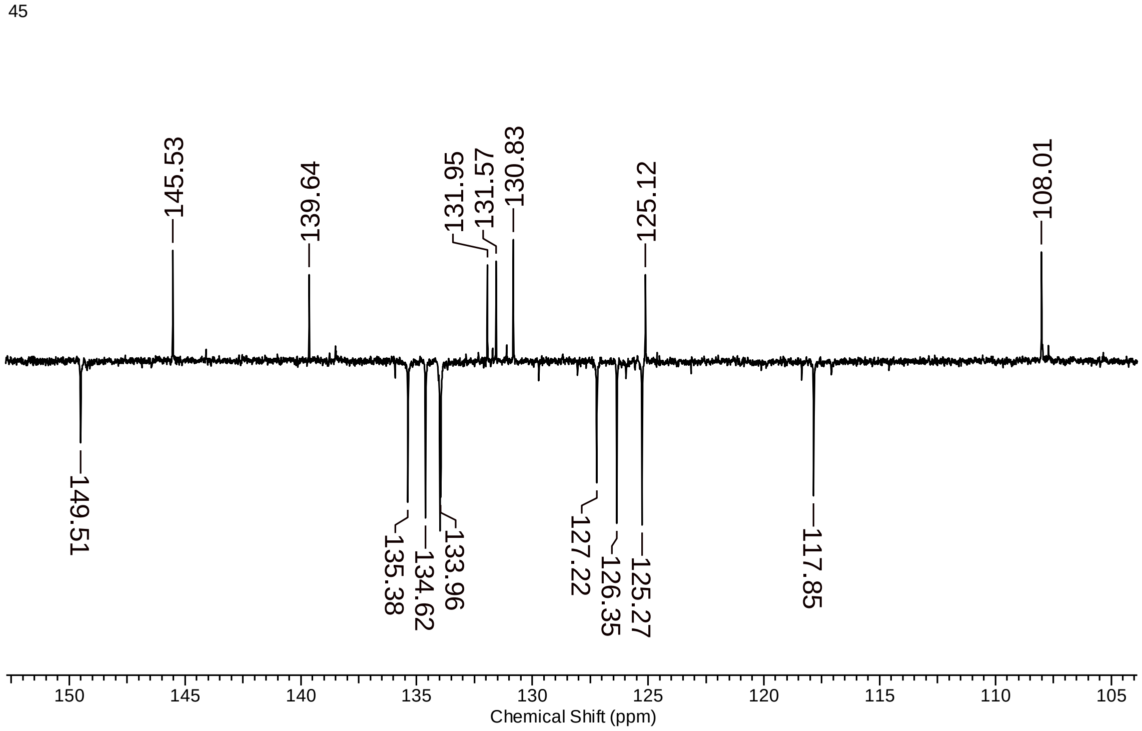


**Figure S37.** Expansion of the $^{13}C$-APT NMR spectrum of derivative **11f** (DMSO-$d_6$, 125.00 MHz).

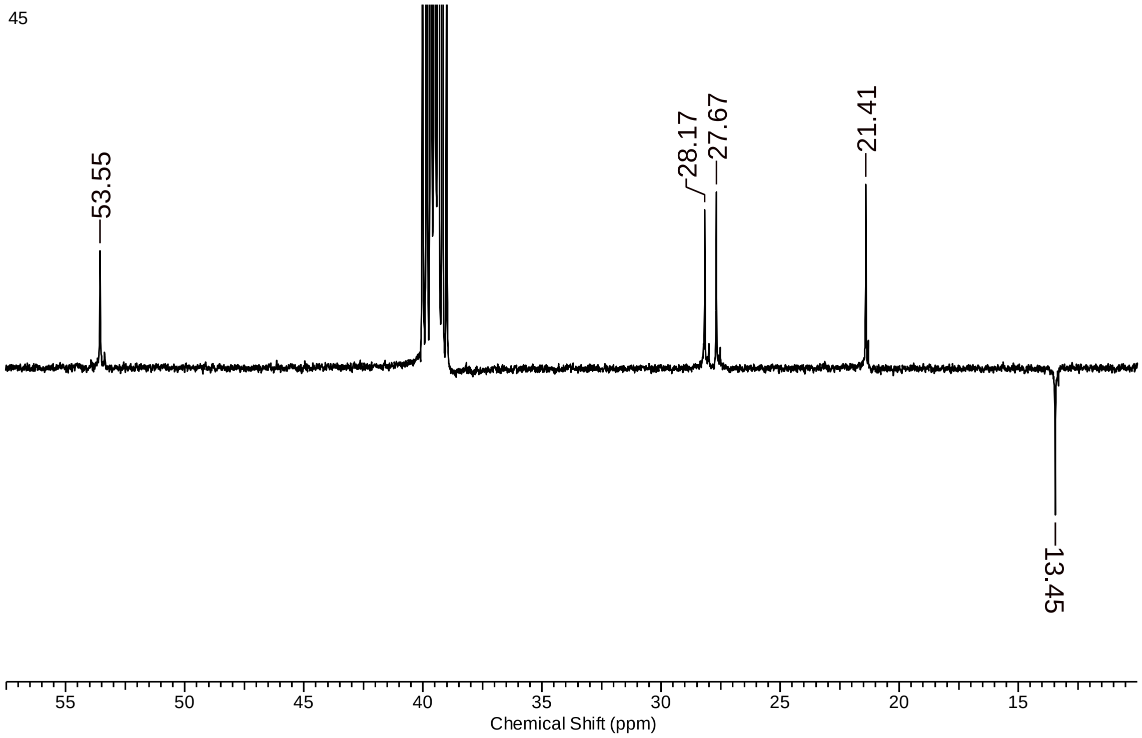


**Figure S38.** Expansion of the $^{13}$C-APT NMR spectrum of derivative **11f** (DMSO-$d_6$, 125.00 MHz).
